# RIT1 regulates mitosis and promotes proliferation by interacting with SMC3 and PDS5 in hepatocellular carcinoma

**DOI:** 10.1186/s13046-023-02892-x

**Published:** 2023-11-29

**Authors:** Yang Su, Hechun Lin, Junming Yu, Lin Mao, Wenjiao Jin, Tengfei Liu, Shuqing Jiang, Yunyu Wu, Saihua Zhang, Qin Geng, Chao Ge, Fangyu Zhao, Taoyang Chen, Ying Cui, Jinjun Li, Helei Hou, Xinli Zhou, Hong Li

**Affiliations:** 1 State Key Laboratory of Systems Medicine for Cancer, Shanghai Cancer Institute, Renji Hospital, Shanghai Jiaotong University School of Medicine, Shanghai, 200032 China; 2 Department of Oncology, Shanghai Tenth People’s Hospital, Tongji University School of Medicine, Shanghai, 200072 China; 3 Department of Oncology, Renji Hospital, Shanghai Jiaotong University School of Medicine, Shanghai, 200127 China; 4 Qidong Liver Cancer Institute, Qidong, 226200 China; 5 Cancer Institute of Guangxi, Nanning, 530021 China; 6 Precision Medicine Center of Oncology, The Affiliated Hospital of Qingdao University, Qingdao, 266003 China; 7 Department of Oncology, Huashan Hospital, Fudan University, Shanghai, 200040 China

**Keywords:** Hepatocellular carcinoma, RIT1, Mitosis, SMC3, PDS5

## Abstract

**Background:**

As a small G protein of Ras family, Ras-like-without-CAAX-1 (RIT1) plays a critical role in various tumors. Our previous study has demonstrated the involvement of RIT1 in promoting malignant progression of hepatocellular carcinoma (HCC). However, its underlying mechanism remains unclear.

**Methods:**

Gene set enrichment analysis (GSEA) was conducted in the TCGA LIHC cohort to investigate the underlying biological mechanism of RIT1. Live cell imaging, immunofluorescence (IF) and flow cytometry assays were used to verify biological function of RIT1 in HCC mitosis. Subcutaneous xenografting of human HCC cells in BALB/c nude mice was utilized to assess tumor proliferation in vivo. RNA-seq, co-immunoprecipitation (Co-IP), mass spectrometry analyses, western blot and IF assays were employed to elucidate the mechanisms by which RIT1 regulates mitosis and promotes proliferation in HCC.

**Results:**

Our findings demonstrate that RIT1 plays a crucial role in regulating mitosis in HCC. Knockdown of RIT1 disrupts cell division, leading to G2/M phase arrest, mitotic catastrophe, and apoptosis in HCC cells. SMC3 is found to interact with RIT1 and knockdown of SMC3 attenuates the proliferative effects mediated by RIT1 both in vitro and in vivo. Mechanistically, RIT1 protects and maintains SMC3 acetylation by binding to SMC3 and PDS5 during mitosis, thereby promoting rapid cell division and proliferation in HCC. Notably, we have observed an upregulation of SMC3 expression in HCC tissues, which is associated with poor patient survival and promotion of HCC cell proliferation. Furthermore, there is a significant positive correlation between the expression levels of RIT1, SMC3, and PDS5. Importantly, HCC patients with high expression of both RIT1 and SMC3 exhibit worse prognosis compared to those with high RIT1 but low SMC3 expression.

**Conclusions:**

Our findings underscore the crucial role of RIT1 in regulating mitosis in HCC and further demonstrate its potential as a promising therapeutic target for HCC treatment.

**Supplementary Information:**

The online version contains supplementary material available at 10.1186/s13046-023-02892-x.

## Introduction

Primary liver cancer is the fourth leading cause of cancer-related deaths globally [1], projected to affect more than one million people annually by 2025. Hepatocellular carcinoma (HCC) is the most common type of primary liver cancer, accounting for 90% of cases [2]. Despite surgical resection being an effective treatment for early-stage HCC, the insidious onset and rapid progression of this malignancy pose challenges in its early diagnosis. Most patients are diagnosed during intermediate or advanced stages, missing the opportunity for surgical intervention, with approximately 50% necessitating systemic treatment. Despite recent advancements in systemic treatment for liver cancer, high rates of recurrence and metastasis coupled with poor response to drug therapies contribute to a persistently high mortality rate for HCC, resulting in a five-year survival rate of only 21% [3]. Therefore, it is of great significance to further elucidate the mechanisms underlying the development and occurrence of HCC and identify new therapeutic targets.

Ras-like-without-CAAX-1 (RIT1) is a member of the RIT subfamily within the Ras superfamily, which includes RIT, RIN, and the Drosophila homolog RIC. It was initially discovered more than 20 years ago [4, 5]. Located on chromosome 1q22, RIT1 serves as a regulatory factor for neuronal cell proliferation, survival, and differentiation [6, 7]. A Rit1 knockout mouse study showed that Rit1 mediates oxidative stress resistance, contributing to cell survival via the p38 MAPK signaling pathway [8]. Multiple RIT1 mutations have been identified in patients with Noonan syndrome (NS) [9, 10]. Aberrant activation and mutations in RAS family genes are commonly observed features in various human malignancies [11]. Somatic mutations and amplifications at the locus of RIT1 have been detected in lung cancer [12–16] as well as bone marrow tumors like chronic myeloid leukemia [17]. In a specific subtype of lung adenocarcinomas, RIT1 has been identified as an oncogene driver capable of inducing transformation in NIH3T3 cells through its mutated forms [16]. Further investigations reveal that mutated RIT1 leads to RIT1 stabilization by escaping LZTR1-mediated degradation and activates MAPK signaling [18]. Consistently, Leukemia-associated RIT1 mutations, which stabilize RIT1 proteins, strongly enhance the self-renewal of hematopoietic stem cells and leukemia progression [19]. In our previous study, we analyze RIT1 mutations in the TCGA LIHC cohort and 21 samples from HCC patients in our laboratory. However, no RIT1 mutations are identified except for one nonfunctional mutation (Q11E) in RIT1 isoform 1 [20]. It is worth noting that RIT1 is frequently amplified in various human cancers including HCC, lung adenocarcinoma, cholangiocarcinoma, uterine carcinosarcoma, breast cancer, and ovarian cancer [15, 21]. Furthermore, high expression of RIT1 has been associated with poor prognosis in patients [20, 22, 23]. Notably, RIT1 is the most common genetic alteration within the RAS family observed in HCC. DNA amplification of RIT1 occurs in approximately 13% of the HCC cohort which emphasizes the significance of studying its amplification and overexpression specifically within HCC [21]. Additionally, we have discovered that hypoxia upregulates RIT1 expression through HIF1-α in HCC. Moreover, overexpression of RIT1 promotes growth as well as migration and invasion capabilities of HCC cells. These findings suggest that RIT1 plays a crucial role in promoting malignant progression of HCC and may serve as a potential molecular marker for HCC [20]. Nevertheless, the precise mechanism by which RIT1 promotes HCC progression remains unclear.

In this study, we demonstrate the crucial role of RIT1 in the mitosis of HCC cells. RIT1 protects and maintains the acetylation of SMC3 through its interaction with PDS5, thereby ensuring rapid mitotic progression in HCC cells and promoting tumor progression. Overall, our findings suggest that targeting RIT1 to induce mitotic catastrophe holds promise as a therapeutic strategy for HCC.

## Materials and methods

### Clinical samples

Two hundred and one human primary HCC tissue specimens were collected with informed consent from the Guangxi Cancer Institute (Nanning, China) and the Qidong Liver Cancer Institute (Qidong, China) between 2001 and 2009. Informed consent was obtained from all patients. All patients received neither chemotherapy nor radiotherapy prior to surgery. This study was approved by the Ethics Committee of Renji Hospital, Shanghai Jiao Tong University School of Medicine, with an approval number KY2021-192-B.

### Cell culture

Human HCC cells MHCC-97H were generously provided by Zhongshan Hospital, Fudan University (Shanghai, China). HCC-LY10 cells were established using the human primary HCC tissues in our laboratory; Huh7 cells were obtained from the Riken Cell Bank (Tsukuba, Japan), while Hep3B and HEK 293T cells were purchased from American Type Culture Collection (Manassas, VA, USA). Cells were cultured in DMEM (BasalMedia Technologies, Shanghai, China) containing 10% FBS (Gibco, New York, USA) and maintained at a temperature of 37 ℃ with a humidified atmosphere containing 5% carbon dioxide. All cell lines were authenticated using STR profiles.

### Plasmid constructs and lentivirus packaging

The human RIT1 coding sequence was amplified by PCR and cloned into the lentiviral expression vector pWPXL (Addgene, Cambridge, MA, USA) and pcDNA3.1-Myc vector. Flag-SMC3 was purchased from Guangzhou Fulengen Co., Ltd (Guangzhou, China). The N-terminal and C-terminal truncations of RIT1were separately amplified and inserted into the pcDNA3.1-Myc vector for Co-IP assay at the XhoI and KpnI sites. Short hairpin RNAs (shRNAs) targeting RIT1, SMC3, and negative control (NC) were obtained from Horizon Discovery (Cambridge, United Kingdom). siRNA oligonucleotides for ESCO1, PDS5A, and PDS5B were purchased from RiboBio (Guangzhou, China). Target sequences of the shRNA and siRNA are listed in Supplemental Table S1. Lentivirus packing and plasmid transfection were conducted following the instructions provided by PolyPlus Transfection (Ilkirch, France).

### Quantitative real-time PCR

Total RNA was extracted from cells using TRIzol Reagent (Invitrogen, California, USA). RNA was then reverse-transcribed into cDNA using a PrimeScript RT-PCR kit (Takara, Japan). Quantitative real-time PCR analyses were conducted using SYBR Premix Ex Taq (Takara, Japan) on a 7500 PCR system according to the manufacturer’s protocol. GAPDH was utilized as an internal control. The primer sequences are listed in Supplemental Table S2.

### Western blot

The extraction of cell and tissue proteins was carried out using T-PER Protein Extraction Reagent (Thermo Fisher Scientific, Massachusetts, USA) supplemented with protease and phosphatase inhibitor (Roche, Basel, Switzerland). Protein quantification was performed by a BCA Protein Assay Kit (Pierce Biotechnology, Rockford, IL, USA). Subsequently, the protein samples were separated by 10% and 8% SDS-PAGE and transferred onto PVDF membranes (Millipore, Bedford, MA, USA). Following blocking with 5% skim milk, the PVDF membranes were incubated overnight at 4 ℃ with a primary antibody. On the following day, the membranes were incubated with a specific HRP-conjugated secondary antibody for 2 h. Visualization of target protein bands was achieved through chemiluminescence detection methods. Details regarding the utilized antibodies can be found in Supplemental Table S3.

### Cell proliferation and colony formation assay

Cell proliferation was assessed using the CCK8 assay (Beyotime, Shanghai, China). The cells were seeded in 96-well plates at varying densities based on the growth characteristics of the cell lines. A fixed volume of CCK8 reagent (10 µL) and medium (90 µL) were added to each well at a predetermined time and incubated for 2 h at 37 °C. The absorbance was measured at 450 nm. For colony formation assays, cells were seeded and cultured for approximately 14 days in six-well plates (ensuring most cell clumps achieved > 50 cells). Subsequently, the cells were then washed with PBS, fixed in neutral phosphate-buffered formalin for 30 min, and stained with crystal violet (Sigma-Aldrich, USA). Each experiment was performed in triplicates.

### Live cell imaging

The progression of cell division was monitored using time-lapse video microscopy. Cells from different groups were seeded in 12-well plates and placed in an incubator equipped with a Nikon (Tokyo, Japan) Biostation Time-lapse system. The imaging field was carefully selected and the focal length adjusted to ensure accurate positioning of the photographs. Images were continuously captured at 30-min intervals for a duration of 10 h.

### Immunohistochemistry

Tissue microarrays (TMA) were prepared for immunohistochemical (IHC) staining. IHC was performed following previously described protocols [24]. Two independent pathologists, who were blinded to the patient’s specific medical features, evaluated the staining results. The IHC results were scored from 0 to 4 according to the percentage of positive cells and staining intensity. A score of 0-2 was considered low protein expression, while a score of 3-4 was considered high protein expression. The antibodies used are listed in Supplemental Table S4.

### Immunofluorescence

Cells were inoculated on Lab-Tek II chamber slides (Thermo Fisher Scientific, USA) and treated with synchronization reagents to gather cells at different cell cycle phases. Subsequently, cells were fixed in 4% paraformaldehyde for 30 min at 25 °C and infiltrated with 0.5% Triton X-100 for 20 min. After blocking with Blocker casein in PBS (Thermo Fisher Scientific, USA) for 30 min, the cells were incubated with primary antibodies overnight at 4 °C. The following day, species-specific secondary antibodies conjugated to Alexa Fluor-546 or -488 were applied to the cells and incubated for 1 h at 25 °C. Cell nuclei were stained using DAPI. Immunofluorescence images were acquired using a confocal microscope (Leica Microsystems, Wetzlar, Germany). The specific antibodies used are listed in Supplemental Table S4.

### Co-immunoprecipitation assay

Cell proteins were harvested using RIPA lysis buffer (Millipore, Bedford, MA, USA) supplemented with protease and phosphatase inhibitors (Roche). Following protein quantification, IgG or specific antibodies were added into the protein lysate and incubated at 4 ℃ under gentle rotation for 16 h. Protein A/G-agarose beads (Abmart, Shanghai, China) were then introduced into the mixture and rotated for an additional 2 h. Subsequently, the beads were washed three times with RIPA lysis buffer and then boiled a protein-loading dye. Finally, the complexes were subjected to western blot analysis.

### Flow cytometry for cell apoptosis and cell cycle analysis

Human HCC cell lines with stable knockdown of RIT1 and SMC3 were used in this study. The cells were collected and fixed in 70% ethanol. The staining reagents consisted of propidium iodide at a concentration of 50 mg/mL, Triton X-100 at a concentration of 0.2%, and RNase A at a concentration of 100 µg/mL. The cell cycle was monitored using a BD LSRII flow cytometer (Becton Dickinson, Franklin Lakes, NJ, USA), and the results were analyzed using ModFit software. For the apoptosis assay, cells were collected and stained with PE and 7-ADD (BD Biosciences, New York, USA) following the manufacturer’s protocol. The percentage of PE (+) cells was measured, and the data were analyzed using the FlowJo software.

### Animal studies

The Huh7 cells (2 × 10^6^), with a stable expression of pWPXL, RIT1, or RIT1 with shSMC3 were collected and subcutaneously inoculated into male BALB/c nude mice (n = 7, 6-8 week-old). After 20 days, the tumor-bearing mice were sacrificed using CO_2_. The tumors were excised and weighed. The tumor volume was calculated using the formula: volume = (length × width^^2^)/2. All experiments were approved by the Animal Care and Use Committee of the Renji Hospital. The Animal Care and Use Committee approved number is R52022-1025.

### Statistical analysis

The statistical analysis was conducted using R programming (https://www.r-project.org/). The data were presented as the means ± standard deviation (SD). Student’s *t*-test was employed for comparing two-group of data. Spearman correlation method was utilized to analyze the correlation between two molecules. Kaplan-Meier estimates were used for survival analysis. Statistical significance was defined as *P* < 0.05.

## Results

### RIT1 regulates mitotic processes in HCC cells

**Fig. 1:**
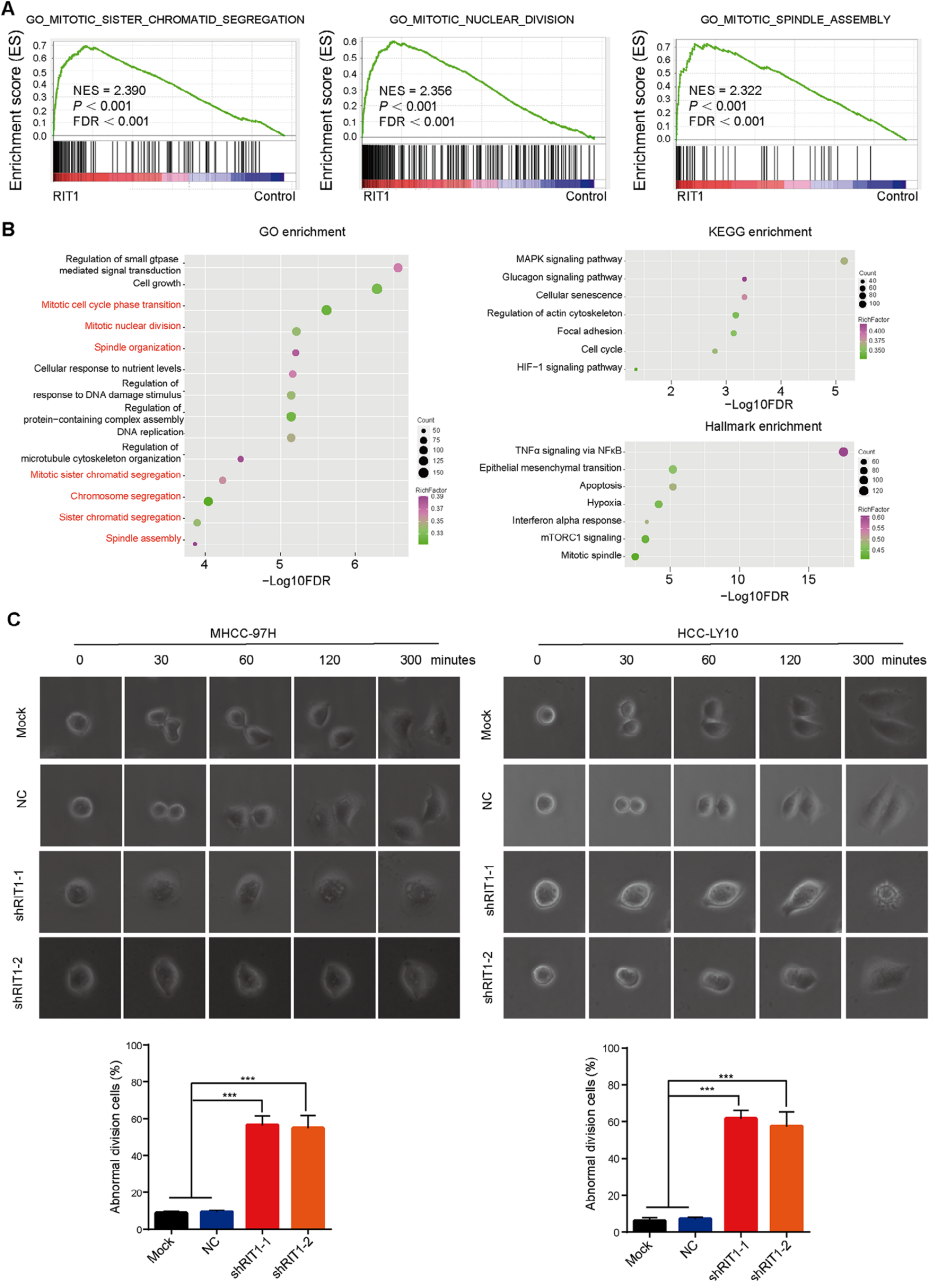
RIT1 is closely associated with the mitotic processes in HCC. **A** Gene set enrichment analysis (GSEA) of a TCGA LIHC cohort with 374 tissues stratified by the mean cut-off value of RIT1 expression. **B** GO, KEGG, and HALLMARK enrichment analysis based on differential genes detected by RNA-Seq in the RIT1 overexpressing and control group of HCC cells. The terms are ordered by -log10 FDR. **C** Comparison of the representative time-lapse images of dynamic division at different time points between RIT1 knockdown and control groups in MHCC-97H and HCC-LY10 cells (upper section). The quantitation histogram showing the percentage of abnormal division cells (lower section). Data are presented as mean ± SD of three independent experiments. *** *P* < 0.001. The* P* values were calculated by unpaired Student’s *t*-test in (**C**)

To investigate the underlying mechanism by which RIT1 promotes HCC progression, we conducted GSEA using the TCGA LIHC database. Our findings revealed a strong correlation between high RIT1 expression in HCC tissues and cell growth and mitotic processes, including sister chromatid segregation, spindle assembly, and nuclear division (Fig. 1A and Supplemental Table S5). We established stably RIT1-overexpressed HCC cells and performed RNA-Seq analysis to identify differential gene expression between RIT1 overexpressed and control groups. Genes showing at least a 1.5-fold difference in expression were selected for further gene enrichment analysis. Gene Ontology (GO) analysis demonstrated that RIT1 overexpression was associated with multiple GO terms related to cell growth and mitotic processes, such as mitotic cell cycle transition, mitotic nuclear division, spindle assembly, and sister chromatid segregation. In addition, KEGG pathway analysis along with hallmark analyses indicated that RIT1 was involved in various signaling pathways including MAPK signaling pathway, epithelial-mesenchymal transition, interferon alpha response, mTORC1 signaling, and hypoxia (Fig. 1B), which is consistent with previous reports [15, 20]. The above analyses of TCGA and RNA-Seq results support that RIT1 expression levels are closely associated with mitotic processes in HCC.

To investigate the biological function of RIT1 in mitosis, we examined the expression of RIT1 protein in various HCC cell lines using western blot (Fig. S1A). MHCC-97H and HCC-LY10 cells were selected for lentiviral transfection to establish stably RIT1-knockdown cell lines. The knockdown efficiency of the RIT1 protein was assessed using qPCR and western blot analysis (Fig. S1B). Subsequently, we performed live cell imaging and dynamically observed the cell division process. The results demonstrated that depletion of RIT1 led to cell division failure, with abnormal phenomena observed in both MHCC-97H and HCC-LY10 cells including cell enlargement, cell vacuolation, binucleated cells, multinucleated cells, and cell death (Fig. 1C). These results suggest a pivotal role for RIT1 in mitosis in HCC.

### Knockdown of RIT1 induces mitotic catastrophe and apoptosis in HCC cells

**Fig. 2:**
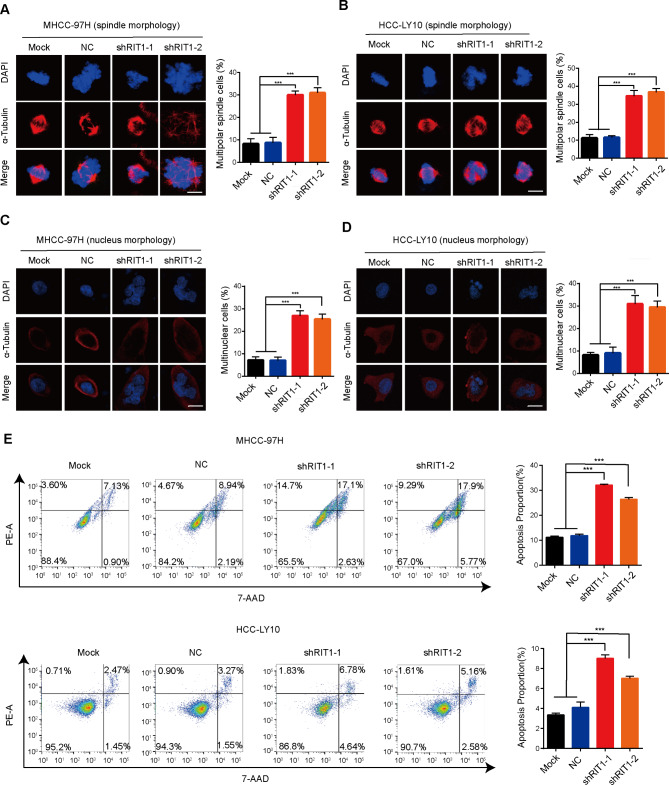
Knockdown of RIT1 induces mitotic catastrophe and apoptosis in HCC cells. **A, B** Representative immunofluorescence staining images of α-tubulin (red) and DAPI (blue) show spindle morphology during mitosis in MHCC-97H (A) and HCC-LY10 cells (B) with RIT1 knockdown and control (left). The quantitation histogram showing the percentage of cells with multipolar spindle (right). **C, D** Representative immunofluorescence staining images of α-tubulin (red) and DAPI (blue) show nucleus morphology in MHCC-97H (**C**) and HCC-LY10 cells (**D**) with RIT1 knockdown and controls (left). The quantitation histogram showing the percentage of cells with multinuclear (right). Cells were enriched in mitosis utilizing nocodazole. Scale bars, 20 μm. **E** Flow cytometry analysis of apoptosis in MHCC-97H and HCC-LY10 cells with RIT1 knockdown and control group (left). The quantitation histogram showing the percentage apoptotic cells (right). Data are presented as mean ± SD of three independent experiments. *** *P* < 0.001. The *P* values were calculated by unpaired Student’s *t*-test

Rapid proliferation due to mitotic dysregulation is a significant feature of cancer, and precise cell cycle progression is essential for proper cell division and proliferation [25]. Given the involvement of RIT1 in mitotic processes and its promotion of HCC growth, we employed flow cytometry to investigate the effect of RIT1 on cell cycle progression. Cell cycle assays revealed an increased percentage of cells in the G2/M phase following RIT1 knockdown, indicating that RIT1 knockdown induced G2/M phase arrest (Fig. S2A). Precisely regulated mitosis ensures proper chromosome segregation, whereas abnormal mitosis can cause mitotic catastrophe, leading to cell death [26]. Subsequently, we examined whether silencing RIT1 affects spindle formation during mitosis in MHCC-97H and HCC-LY10 cells. The immunofluorescence (IF) staining results showed that silencing RIT1 caused a significantly increased multipolar spindle cells (Fig. 2A, B), which are hallmarks of mitotic catastrophe. To confirm the mitotic catastrophe caused by RIT1 interference, we further observed the morphological characteristics of cell nuclei. We found that RIT1 knockdown resulted in obvious multinucleated, heterogeneous nuclei in MHCC-97H and HCC-LY10 cells (Fig. 2C, D). Furthermore, according to the 7-ADD/propidium iodide staining analysis, RIT1 knockdown induced significant apoptosis in MHCC-97H and HCC-LY10 cells (Fig. 2E). These data suggest that knockdown of RIT1 causes cell cycle arrest, triggers mitotic catastrophe, and promotes apoptosis in HCC cells.

### RIT1 interacts with SMC3 in HCC cells

**Fig. 3:**
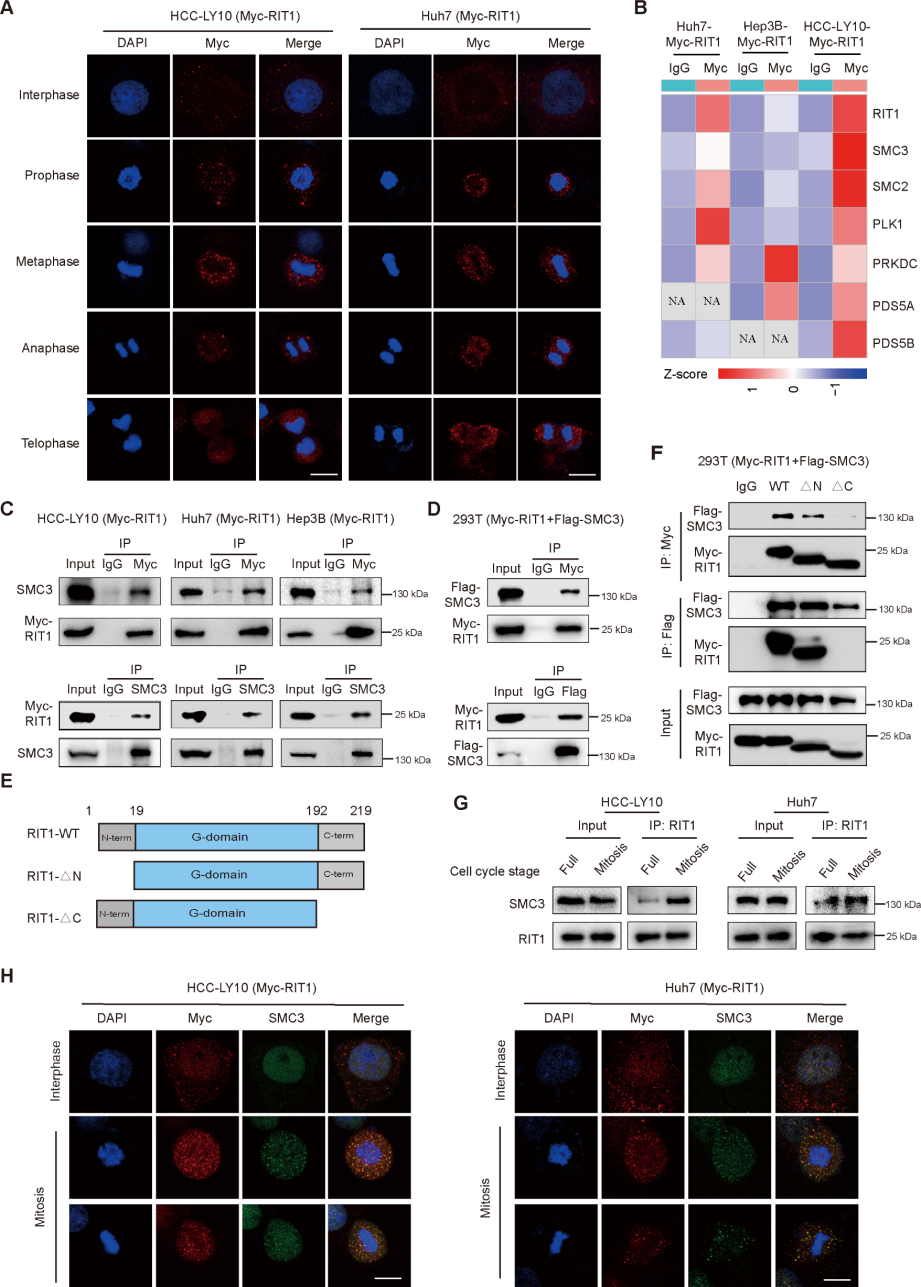
RIT1 interacts with SMC3 in HCC cells. **A** Immunofluorescence staining of Myc-tag (red) and DAPI (blue) shows the distribution of RIT1 at different cell cycle phases in HCC-LY10 and Huh7 cells transfected with Myc-RIT1 plasmid. Scale bars, 20 μm. **B** Heat map of mitotic chromosome segregation-related proteins across Myc-RIT1-precipitated compared to IgG-precipitated group in HCC-LY10, Huh7, and Hep3B cells transfected with Myc-RIT1 plasmid. **C** Co-IP analysis of RIT1 and SMC3 in HCC-LY10, Huh7 and Hep3B cells transfected with Myc-RIT1 plasmid. Cell lysates from indicated cells were immunoprecipitated using an anti-Myc antibody (upper section) and an anti-SMC3 antibody (lower section). **D** Interaction of RIT1 and SMC3 was analyzed by Co-IP analysis in 293T cells co-transfected with Myc-RIT1 and Flag-SMC3 plasmids. Cell lysates were immunoprecipitated using an anti-Myc antibody (upper section) and an anti-Flag antibody (lower section). **E** Schematic presentation of structural domain of RIT1. **F** Co-IP analysis of the indicated RIT1 truncation and wild-type with SMC3 for their interaction position in 293T cells co-transfected with Myc-RIT1 (WT, ΔN or ΔC) and Flag-SMC3 plasmids. **G** Co-IP analysis of RIT1 and SMC3 in Huh7 and HCC-LY10 cells with RIT1 overexpression during asynchronous and synchronized to mitosis (Full indicates asynchronization treatment; Mitosis indicates cells were enriched in mitosis). **H** Colocalization of RIT1 and SMC3 during interphase and different phases of mitosis was analyzed by co-immunofluorescence staining (Myc-RIT1: red, SMC3: green, DAPI: blue) in HCC-LY10 (left) and Huh7 (right) cells transfected with Myc-RIT1 plasmid. Scale bars, 20 μm

Previous studies on the oncogenic mechanism of RIT1 have focused mainly on its regulation of the RAS/MAPK pathway. A recent study revealed that the RIT1 is diffusely distributed in the cytoplasm during mitosis and interacts with MAD2, a component of the spindle assembly checkpoint (SAC), to negatively regulate SAC activity [27]. To explore the underlying molecular mechanism by which RIT1 regulates mitosis in HCC, we transfected Myc-tagged RIT1 plasmids into Huh7, Hep3B, and HCC-LY10 cells and detected RIT1 distribution during different cell cycle stages by immunofluorescence staining with an anti-Myc antibody. The results demonstrated that RIT1 exhibited diffuse cytoplasmic localization during interphase and gradually accumulated around the chromosomes during mitosis (Fig. 3A and Fig. S3A). These results further suggested that RIT1 is closely associated with mitotic processes in HCC.

Mitosis is a process involving multiple protein interactions. Based on the subcellular localization pattern of RIT1 during mitosis, we hypothesized that RIT1 may affect the mitotic process of HCC cells by interacting with regulatory proteins involved in mitosis. Co-immunoprecipitation (Co-IP) combined with mass spectrometry (MS) was performed to screen for potential RIT1 interacting proteins in HCC-LY10, Huh7, and Hep3B cells transfected with Myc-tagged RIT1 plasmid. The screening for interacting proteins was based on their abundance in the Myc-RIT1 group, which was three times higher than that in the control IgG group. By intersecting the results from all three cell lines (Fig. S4A), we observed that MAD2 protein was not detected in our MS analysis; however, several proteins associated with regulation of mitotic chromosome separation, including structural maintenance of chromosome 3 (SMC3), structural maintenance of chromosome 2 (SMC2), polo like kinase 1 (PLK1), and protein kinase, DNA-activated, catalytic subunit (PRKDC) proteins, were identified in the intersection of three cell lines. In addition, we found that PDS5A and PDS5B, chromosomal cohesin regulatory subunits, were present at two cell intersections (Fig. 3B).

We selected SMC3, SMC2, PLK1, and PRKDC, which were present at the three-cell intersections, for further validation. Co-IP and western blotting analyses revealed that only SMC3 interacted with RIT1 in HCC-LY10, Huh7, and Hep3B cells (Fig. 3C and Fig. S4B). The interaction between RIT1 and SMC3 was further verified in 293T cells co-transfected with Myc-RIT1 and Flag-SMC3 plasmids (Fig. 3D). To determine which domain of RIT1 interacts with SMC3, wild-type or truncated Myc-RIT1 was transfected with Flag-SMC3 plasmids in 293T cells and immunoprecipitation and western blot were performed using anti-Myc and anti- Flag antibodies, respectively. The results showed that the wild-type and N-terminal deletion constructs of Myc-RIT1 could bind to SMC3, whereas the C-terminal deletion construct could not, indicating that the binding sites were present in the C-terminal domain of RIT1 (Fig. 3E, F). Moreover, Co-IP analyses with cells asynchronized or synchronized to mitosis showed that the interaction between RIT1 and SMC3 was significantly enhanced during mitosis (Fig. 3G). IF assays showed apparent colocalization of RIT1 with SMC3 proteins during mitosis in HCC-LY10, Huh7, and Hep3B cells (Fig. 3H and Fig. S5A). These results suggest that RIT1 interacts with SMC3, which is essential for mitosis in HCC.

### SMC3 is essential for RIT1-mediated cell proliferation in HCC

**Fig. 4:**
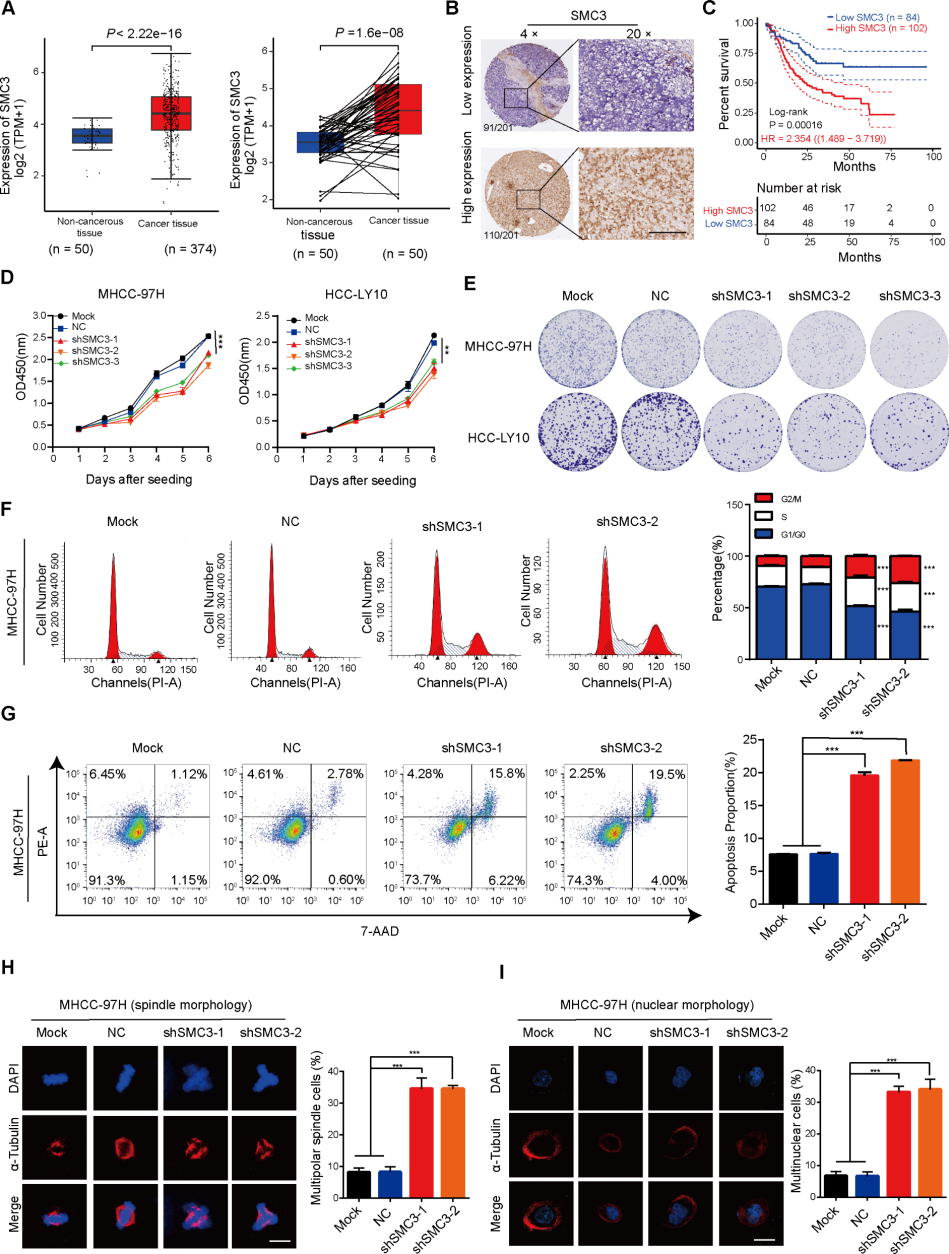
SMC3 is upregulated in HCC and contributes to HCC proliferation. **A** mRNA expression of SMC3 in 374 HCC tissues and 50 non-cancerous tissues in the TCGA LIHC database (left). mRNA expression of SMC3 in 50 pairs of HCC tissues and adjacent non-cancerous tissues in the TCGA LIHC database (right). **B** Representative IHC staining images of high and low SMC3 expression in 201 HCC tissue samples, including 110 cases of high expression and 91 cases of low expression based on the IHC staining score; scale bars, 200 μm. **C** Kaplan-Meier analysis showing the overall survival of 186 patients with HCC with high-SMC3 and low-SMC3 expression; HR, Hazard Ratio, *P* = 0.00016. **D** CCK8 assay for MHCC-97H and HCC-LY10 cells with SMC3 knockdown and control. **E** Colony formation assay of MHCC-97H and HCC-LY10 cells with SMC3 knockdown and control. **F** Flow cytometry analysis of the cell cycle of MHCC-97H and HCC-LY10 cells with SMC3 knockdown and control (left). Quantitation histograms showing the percentage of cells in different phases of the cell cycle (right panel). **G** Flow cytometry analysis of apoptosis in MHCC-97H cells with SMC3-knockdown and control cells (left). Quantitation histogram showing the percentage of apoptotic cells (right). **H** Representative immunofluorescence staining images of α-tubulin (red) and DAPI (blue) showing spindle morphology during mitosis in MHCC-97H cells with SMC3 knockdown and control(left). The quantitation histogram showing the percentage of cells with multispindle polar (right). Cells were enriched in mitosis using nocodazole. Scale bars, 20 μm. **I** Representative immunofluorescence staining images of α-tubulin (red) and DAPI (blue) show nucleus morphology in MHCC-97H cells with SMC3 knockdown and control (left). The quantitation histogram showing the percentage of cells with multinuclear (right). Scale bars, 20 μm. Data are presented as the mean ± SD of three independent experiments. ** *P* < 0.01, *** *P* < 0.001. The *P* values were calculated by paired Student’s *t*-test in (**A**), log-rank test in (**C**), and unpaired Student’s *t*-test in (D-I)

Due to the interaction between RIT1 and SMC3, we hypothesized that RIT1 might play a regulatory role in mitosis through SMC3 in HCC cells. To investigate the role of SMC3 in RIT1-mediated HCC progression, we analyzed the mRNA expression of SMC3 in the TCGA LIHC database. As shown in Fig. 4A, SMC3 expression was significantly higher in HCC tissues than in non-cancerous tissues. We further examined the protein expression of SMC3 in 36 pairs of HCC tissues and matched non-cancerous tissues from our lab using western blot analysis. Consistent with the results of TCGA dataset analysis, SMC3 was highly expressed in HCC tissues compared with that in non-cancerous tissues (Fig. S6A, B). Immunohistochemistry (IHC) staining for SMC3 was performed on tissue samples from 201 patients with HCC, and patients were divided into high-SMC3 expression group (110 cases) and low-SMC3 expression group (91 cases) according to their IHC staining scores (Fig. 4B). Kaplan-Meier survival analysis showed that high SMC3 expression in patients with HCC was associated with a short survival time (Fig. 4C). These results indicate that SMC3 is highly expressed in HCC and is associated with a poor prognosis.

To further investigate the role of SMC3 in HCC cells, we examined the expression of SMC3 protein in various HCC cell lines and knocked down SMC3 via shRNA in MHCC-97H and HCC-LY10 cells and verified the silencing efficiency using western blotting (Fig. S6C, D). Similar effects were observed after SMC3- and RIT1-knockdown in HCC cells. SMC3 knockdown significantly inhibited proliferation and colony formation of MHCC-97H and HCC-LY10 cells (Fig. 4D, E and Fig. S6E) and led to G2/M phase arrest and apoptosis (Fig. 4F, G). Moreover, the knockdown of SMC3 induced mitotic catastrophe, multinucleated and heterogeneous nuclei, and multipolar spindles (Fig. 4H, I and Fig. S6F, G).

**Fig. 5:**
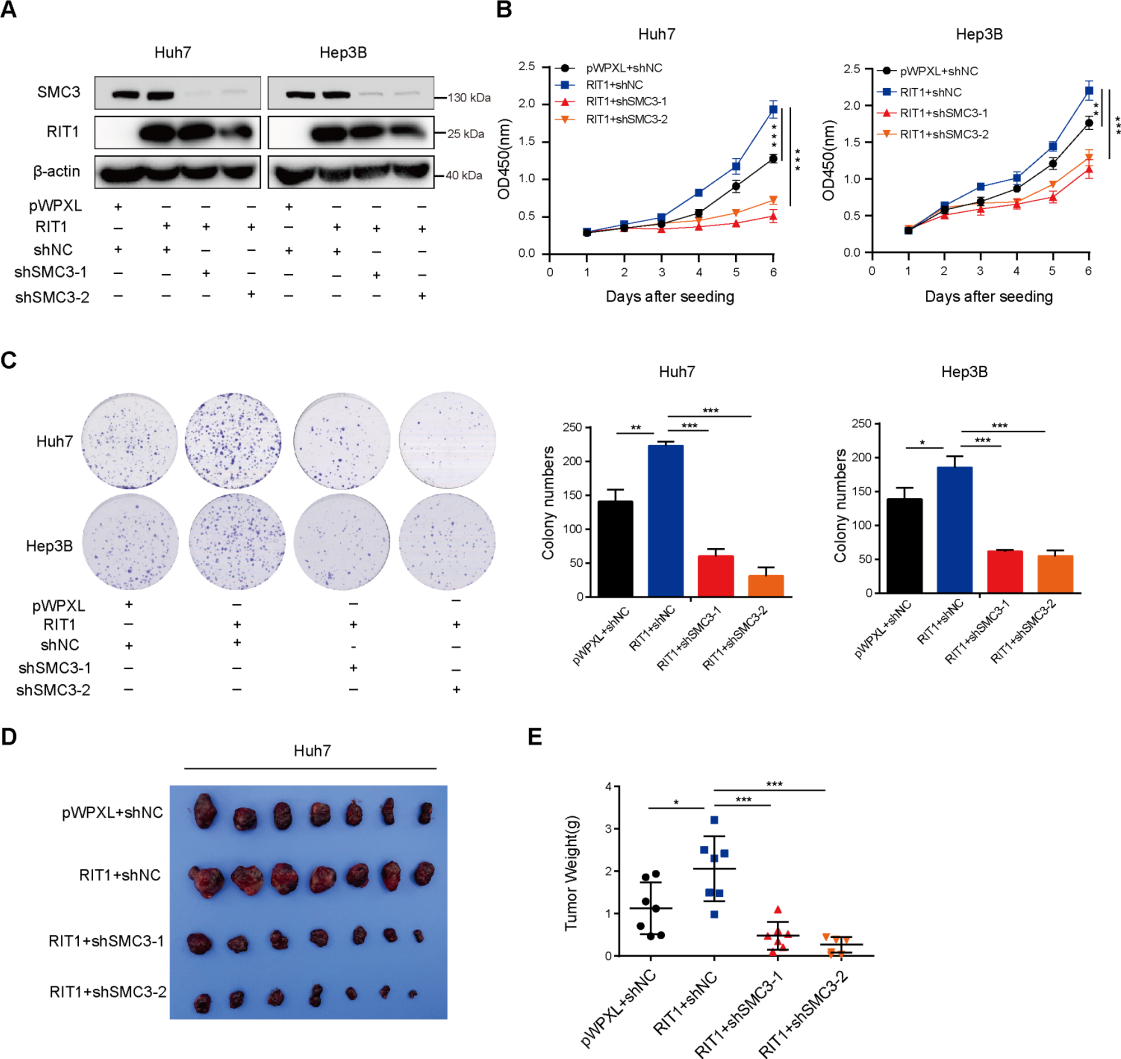
Knockdown of SMC3 inhibits the pro-proliferative effect of RIT1 in HCC cells. **A** Western blot analysis of RIT1 and SMC3 protein expression after silencing SMC3 in RIT1-overexpressing Huh7 and Hep3B cells. **B** CCK8 assay was performed to examine proliferation after silencing SMC3 in RIT1-overexpressing Huh7 and Hep3B cells. **C** Representative images of colony formation assays for RIT1-overexpressing Huh7 and Hep3B cells with SMC3 knockdown (left panel). Quantitation histogram showing colony numbers (right). **D** Images of xenograft tumors of RIT1-overexpressing Huh7 cells or control cells with SMC3 knockdown. **E** Quantification of tumor weight (g) (n = 7 per group). Data are presented as the mean ± SD of three independent experiments. * *P* < 0.05, ** *P* < 0.01, *** *P* < 0.001. The P values were calculated by unpaired Student’s *t*-test for (**B, C, E**)

Given that RIT1 interacts with SMC3 and is co-localized during mitosis, it is reasonable to speculate that RIT1 might promote HCC progression by regulating SMC3. We silenced SMC3 in RIT1-overexpressed Huh7 and Hep3B cells (Fig. 5A). CCK8 and colony formation assays showed that silencing SMC3 attenuated the proliferative effect caused by RIT1 overexpression (Fig. 5B, C). SMC3 knockdown also reversed the tumor-promoting effects of RIT1 in vivo in mouse subcutaneous tumor experiments (Fig. 5D, E). Overall, these data suggest that RIT1 promotes HCC cell proliferation by interacting with SMC3.

### RIT1 affects SMC3 acetylation during mitosis

**Fig. 6:**
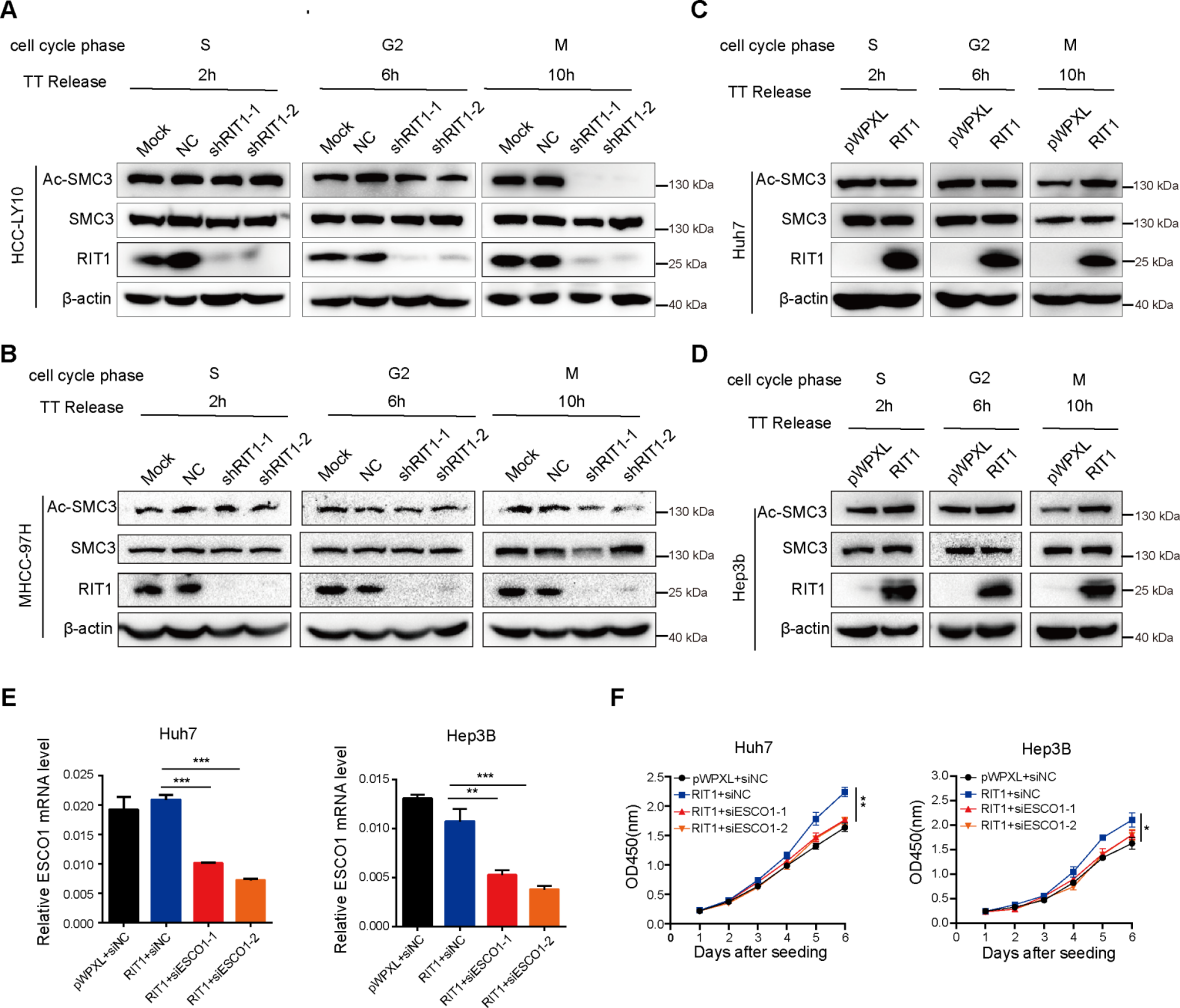
RIT1 affects the acetylation of SMC3 during mitosis. **A, B** Western blot analysis of acetylated-SMC3, total SMC3, and RIT1 protein levels during different cell phases in MHCC-97H (**A**) and HCC-LY10 cells (**B**) with RIT1 knockdown. **C, D** Western blot analysis of acetylated-SMC3, total SMC3, and RIT1 protein levels during different cell phases in Huh7 (**C**) and Hep3B (**D**) cells with RIT1 overexpression. Cells were enriched in different cycle phases by releasing from the TT block for the indicated time. **E** qPCR assays of ESCO1 mRNA expression following si-ESCO1 transfection in RIT1-overexpressed Huh7 and Hep3B cells. **F** The cell proliferation ability was examined using CCK8 assay in RIT1-overexpressing Huh7 and Hep3B cells with ESCO1 knockdown. Data are presented as mean ± SD of three independent experiments. * *P* < 0.05, ** *P* < 0.01, *** *P* < 0.001. The *P* values were calculated by unpaired Student’s *t*-test in (**E, F**)

SMC3 is a member of the cohesin core subunit that ensures accurate chromosomal segregation. Establishing chromosome adhesion depends on the acetylation of K105 and K106 in the SMC3 head domain [28]. To further investigate how RIT1 regulates SMC3 and thus influences HCC cell mitosis, we detected the effect of RIT1 on SMC3 mRNA expression by qPCR and found that RIT1 did not affect the mRNA expression of SMC3 (Fig. S7A, B). Then, we examined the protein levels of total SMC3 and SMC3 acetylation in HCC cells with altered RIT1 expression. Protein samples were collected at different stages of the cell cycle after release from double thymidine block for western blot analysis. The results showed that RIT1 significantly affected the acetylation level of SMC3 during mitosis but not in the S and G2 phases. Knockdown of RIT1 decreased the acetylation level of SMC3, whereas overexpression of RIT1 increased the acetylation level of SMC3. RIT1 did not affect the total protein expression of SMC3 (Fig. 6A-D). These results suggest that RIT1 may regulate mitotic progression in HCC cells by influencing the acetylation level of SMC3 during mitosis. To further validate this result, we knocked down ESCO1, an SMC3 acetyltransferase, in RIT1 overexpressed HCC cells (Fig. 6E) and observed changes in their proliferative capacity. CCK8 experiments demonstrated that ESCO1 knockdown reversed the enhanced proliferative capacity induced by RIT1 overexpression (Fig. 6F). This evidence reveals that RIT1 exerts a pro-oncogenic effect by affecting the acetylation level of SMC3 during mitosis.

### RIT1 protects the acetylation of SMC3 by binding to PDS5

**Fig. 7:**
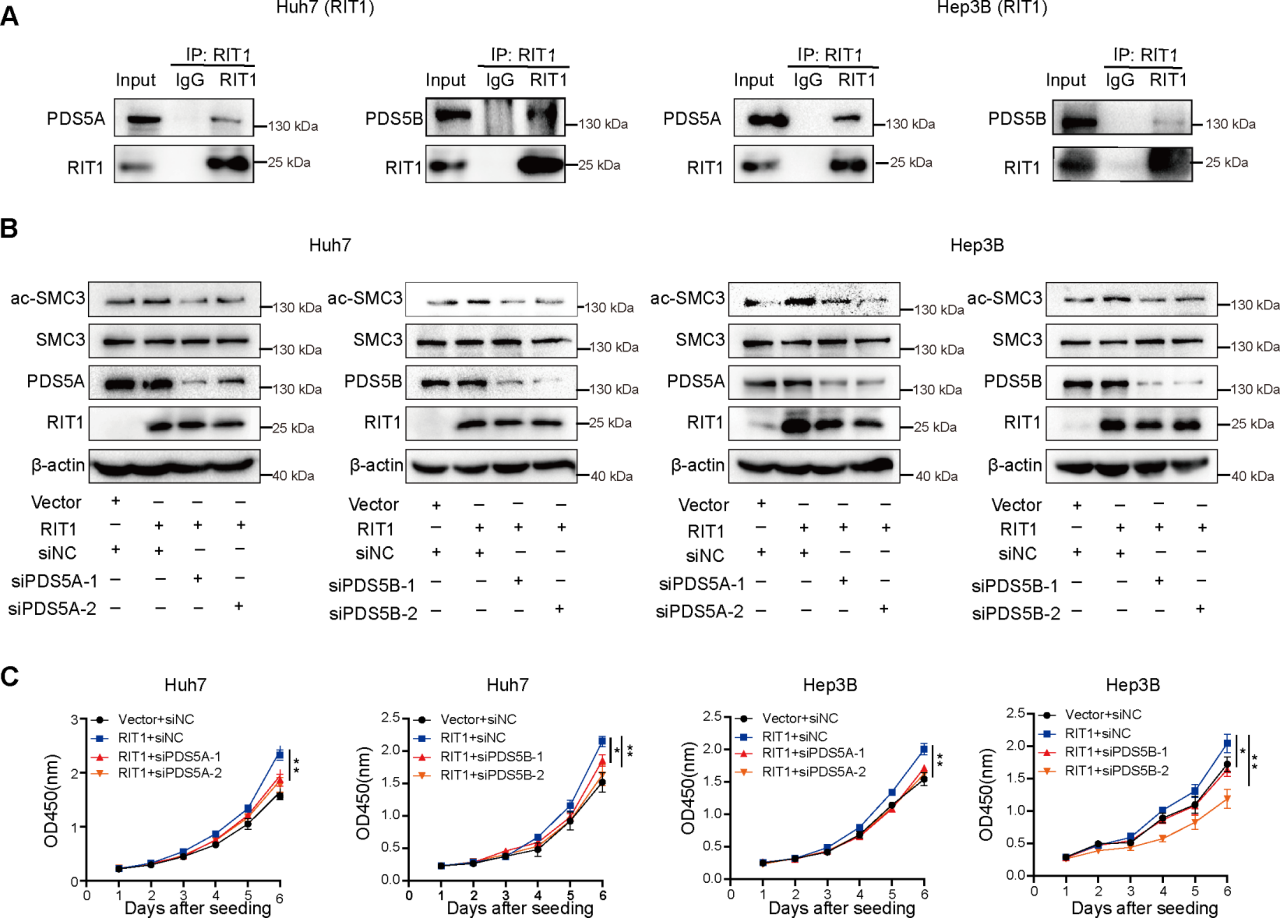
RIT1 promotes HCC proliferation by binding to PDS5 to protect the acetylation of SMC3. **A** Co-IP analysis of RIT1 and PDS5 in Huh7 and Hep3B cells with RIT1 overexpression. **B** Western blot analysis of acetylated-SMC3, total SMC3, PDS5A/B, and RIT1 protein upon knockdown of PDS5A/5B in RIT1-overexpressing Huh7 and Hep3B cells. **C** The cell proliferation ability was examined using CCK8 assay upon knockdown of PDS5A/5B in RIT1-overexpressed Huh7 and Hep3B cells. Data are presented as mean ± SD of three independent experiments. * *P* < 0.05, ** *P* < 0.01. The *P* values were calculated by unpaired Student’s *t*-test in (**C**)

Next, we investigated the regulatory mechanism by which RIT1 affects the acetylation of SMC3. Considering that RIT1 concentrates around the chromosome during mitosis and interacts with SMC3, we hypothesized that RIT1 might play a regulatory role in SMC3 acetylation during mitosis by influencing proteins or kinases involved in SMC3 acetylation regulation. qPCR analysis showed that RIT1 did not affect ESCO1 expression (Fig. S8A, B), and no SMC3 acetyltransferase was detected in the MS results for the Co-IP products mentioned above. However, our MS results indicated an interaction between RIT1 and PDS5 (Fig. 3B). PDS5 is an essential regulatory subunit of cohesin that maintains a dynamic balance between cohesin loading and unloading by binding to sororin and wings apart-like protein (WAPL) homolog proteins [29]. In addition, PDS5 can protect and maintain SMC3 acetylation during mitosis [30]. Therefore, we speculated that RIT1 might regulate the acetylation of SMC3 via PDS5. PDS5 has two subunits: PDS5A and PDS5B. Co-IP and western blot analyses revealed an interaction between RIT1 and PDS5A/B (Fig. 7A), while overexpression or knockdown of RIT1 had no effect on PDS5 expression (Fig. S9A, B). We silenced both PDS5A/B in RIT1 overexpressed HCC cells and examined the total SMC3 and acetylated protein expression. The results showed that the upregulation of SMC3 acetylation induced by RIT1 overexpression was suppressed by PDS5A/B knockdown during mitosis, whereas total levels of SMC3 protein remained unchanged (Fig. 7B). The PDS5A/B knockdown attenuated the enhanced growth capacity induced by RIT1 overexpression (Fig. 7C).

In addition, we further explored whether PDS5 expression affects the interaction between RIT1 and SMC3. Co-IP results demonstrated that knockdown of PDS5 did not affect the binding of RIT1 to SMC3 (Fig. S10A).

These results indicated that RIT1 may function as a molecular scaffold to protect SMC3 acetylation by binding to PDS5.

#### RIT1 expression positively correlates with PDS5 and SMC3 in HCC tissues

**Fig. 8:**
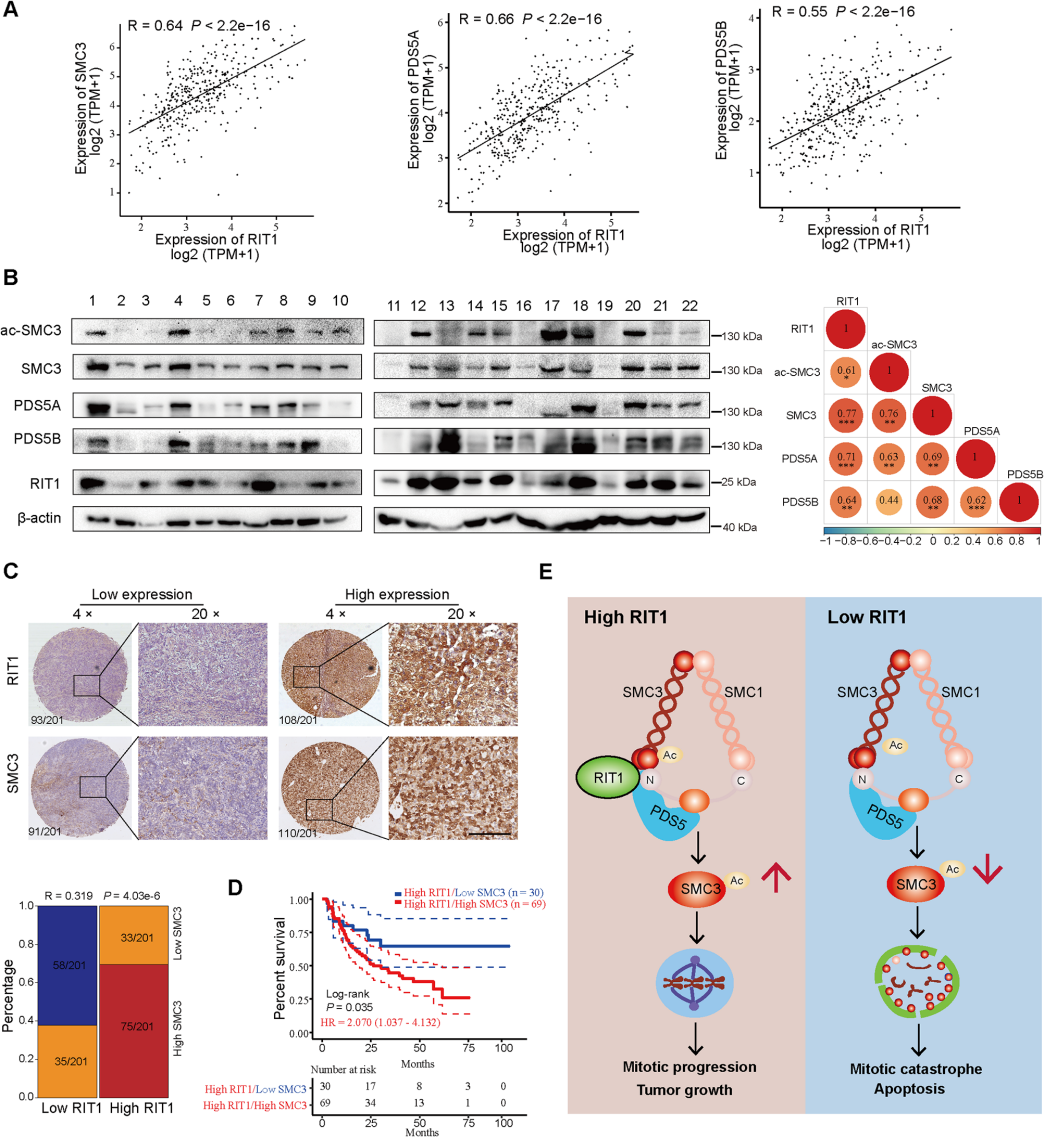
RIT1 expression positively correlates with PDS5 and SMC3 in HCC tissues. **A** Correlation of mRNA level of RIT1 with PDS5A/B and SMC3 in TCGA LIHC dataset. **B** Western blot analysis of RIT1, PDS5A/B, acetylated-SMC3, and total SMC3 in HCC tissues from our lab (left). Spearman correlation analysis was performed between RIT1 and PDS5A/B, acetylated-SMC3, total SMC3 expression (right). **C** Representative IHC staining images of concurrent high or low RIT1/SMC3 expression in 201 HCC tissue samples. Spearman correlation analysis of RIT1 and SMC3 expression was performed (R = 0.319, *P* < 0.001). Scale bars, 200 μm. **D** Kaplan-Meier survival curves for 99 HCC patients with RIT1 high expression classified by high or low expression of SMC3 expression according to IHC results; HR, Hazard Ratio. **E** A schema showing that RIT1 regulates mitosis and promotes proliferation by interacting with SMC3 and PDS5 in HCC. With a high level of RIT1 in HCC, RIT1 binds with PDS5 and SMC3, which protects and maintains the acetylation of SMC3 during mitosis. HCC cells undergo successful and rapid mitosis, leading to tumor growth in HCC (left). A low level of RIT1 in HCC or knockdown of RIT1 leads to a reduction of the acetylation level of SMC3, resulting in mitotic catastrophe in HCC (right)

Given that RIT1 protects the acetylation of SMC3 by binding to PDS5, we analyzed the correlation between RIT1 expression and SMC3 as well as PDS5A/B in HCC tissues. The mRNA level of RIT1 was positively correlated with SMC3 and PDS5A/B expression in the TCGA LIHC dataset (Fig. 8A). Notably, the protein levels of acetylated SMC3, total SMC3, PDS5A/B, and RIT1 were also positively correlated in HCC tissues (Fig. 8B). IHC analysis further confirmed a positive correlation between RIT1 and SMC3 expression in HCC tissues (Fig. 8C). We then divided the patients with high RIT1 expression into two groups according to SMC3 expression levels and observed that patients with high expressions of both RIT1 and SMC3 had a worse prognosis compared to those with high RIT1 but low SMC3 expression (Fig. 8D). These results further conformed that RIT1 expression closely correlated to SMC3 and PDS5A/B expression, and RIT1 exerted its pro-proliferative function via SMC3 in HCC. Our data highlight the significance of the RIT1/PDS5/SMC3 axis in promoting HCC progression and suggest that RIT1 may serve as a biomarker and potential target for HCC diagnosis and therapy (Fig. 8E).

## Discussion

Uncontrolled proliferation and division of cancer cells require the efficient regulation of a variety of associated proteins. Our investigations have unveiled the pivotal role of RIT1/PDS5/SMC3 as a critical regulator in HCC mitosis. During this process, RIT1 protects and maintains SMC3 acetylation by binding to PDS5; disruption of this mechanism leads to substantial mitotic catastrophe, consequently impeding HCC progression. Our study provided novel insights into the involvement of RIT1 in HCC advancement and suggested that developing therapeutics targeting RIT1 to hinder mitosis holds great promise.

RIT1 plays a crucial role in various tumors; however, studies on the carcinogenic mechanisms of RIT1 have primarily focused on the RAS/MAPK pathway. A recent study reported that RIT1 promotes HCC progression by inducing angiogenesis through the MEK/ERK/EIF4E/HIF1-α/VEGFA axis [21]. In this study, bioinformatics analysis reveals a close association between RIT1 and mitotic processes in HCC. Mitotic dysregulation is a principal biological characteristic of tumor cells [31] that undergo rapid cell division by regulating mitosis-related signals. Targeting mitosis has gained significant attention in developing antitumor drugs [32]. Numerous drugs targeting different aspects of mitosis, including centrosome replication, spindle assembly, and chromosome separation, have been developed and tested in clinical trials. However, their widespread application is limited due to severe side effects [33]. A notable feature of liver cells exhibit binucleated or multinucleated cells, with approximately 30-40% of liver cells in adult humans being binucleated while maintaining normal physiological functions [34]. The formation of binucleated cells primarily results from failed cytokinesis. However, most liver cancer cells are diploid, indicating their proliferation heavily relies on cell division [35, 36]. Considering the tolerance of normal liver cells toward binucleation or multinucleation, inhibiting mitosis may effectively impede the growth of liver cancer cells without affecting normal liver function [37]. Therefore, a comprehensive investigation into the key regulatory proteins and their mechanisms of action in mitosis holds significant importance in identifying new strategies for HCC treatment. Our findings demonstrated that knockdown of RIT1 hampers mitosis in HCC cells, triggers mitotic catastrophe, and impedes HCC proliferation. Mitotic catastrophe serves as an intrinsic anticancer mechanism that upholds genome stability by driving cells with defective or failed mitosis to death via necrosis, apoptosis, and senescence [26]. Given that most cancer cells exhibit some degree of aneuploidy, they are more susceptible to death after mitotic damage compared to non-transformed cells. Therefore, induction of mitotic catastrophe is a promising strategy for cancer treatment [38].

Mitosis is a complex biological process that requires the involvement of a series of proteins. The SMC proteins are essential for sister chromatid cohesion and precise segregation during mitosis [39]. Our study revealed that RIT1 interacts with SMC3 via its C-terminal domain. SMC3 is a core component of the cohesin complex, a ring-shaped protein complex composed of SMC1, SMC3, Scc1 (Mcd1 or Rad21), and Scc3 (SA1 and SA2). Cohesins hold significant importance in chromosome segregation, DNA repair, and transcriptional regulation [40]. Previous investigations have demonstrated that the upregulation of the SMC family occurs in various malignancies and contributes to tumor progression [41, 42]. The mRNA levels of SMC3 are elevated in approximately 70% of colon cancer samples, and overexpression of SMC3 promotes fibroblast transformation [43]. A study on lung cancer indicated that hydrogen inhibits lung cancer progression by downregulating SMC3 [44]. Our data exhibited high expression levels of SMC3 in HCC tissues, which correlated with poor prognosis-a finding consistent with a previous report [45]. We also observed a positive correlation between the expression of RIT1 and SMC3 and their co-high expression is associated with a poorer prognosis. Knockdown of SMC3 resulted in similar functional effects as knockdown of RIT1, including inhibition of proliferation, cell cycle arrest, mitotic catastrophe, DNA damage, and apoptosis in HCC cells. Furthermore, knockdown of SMC3 suppressed the proliferative effects mediated by RIT1 in HCC cells. These findings suggest that SMC3 plays an essential role in the regulation of mitosis and progression of HCC mediated by RIT1.

Acetylation of SMC3 plays a vital role for cohesin in facilitating the stable wrapping of sister chromatids by cohesin, thereby ensuring accurate chromosome segregation. Our findings indicated that the expression level of RIT1 affects the acetylation level of SMC3 during mitosis. Our MS results revealed a specific interaction between RIT1 and PDS5, a regulatory subunit of cohesin involved in modulating its loading and unloading through binding to sororin and WAPL. Furthermore, previous studies have reported that PDS5 protects SMC3 acetylation by ESCO1 and prevents its deacetylation by Hos1/HDAC8 through binding to RAD21 in the N-terminal nucleotide-binding domain of SMC3 [30]. We further demonstrated that RIT1 protects and maintains SMC3 acetylation by interacting with PDS5 during mitosis, while knockdown of PDS5 attenuates the pro-proliferative effect induced by RIT1 overexpression. Additionally, our results suggested that PDS5 does not influence the interaction between RIT1 and SMC3. However, further investigation is required to elucidate the regulatory networks involving RIT1, PDS5, and SMC3.

Our study demonstrated that RIT1 plays a crucial role in promoting proper mitotic progression in HCC cells, and the knockdown of RIT1 leads to mitotic catastrophe. It is worth noting that a recent research has shown that RIT1 mutation negatively regulates the SAC by binding to MAD2, thereby accelerating mitosis with some mitotic errors in U2OS and HeLa cells [27]. Additionally, Vichas et al. found that the RIT1^M90I^ mutation attenuates SAC in lung adenocarcinoma, making it vulnerability to Aurora kinase inhibitors [13]. The relationship between mitotic disorders and tumor development is complex. Although multiple mechanisms during mitosis can lead to chromosomal loss and gain, and aneuploidy increases the probability of tumor occurrence and formation [46, 47]. However, aneuploidy has also been reported to antagonize tumorigenesis, suggesting that it alters the path of tumor development [48]. Multiple factors, including cell type, genetic background, and the environment of different tissues, could affect the outcome of aneuploidy [48, 49]. Defects in the regulation of mitosis and DNA damage result in mitotic failure and, ultimately, mitotic catastrophe [26]. The rate of errors in chromosomal segregation determines whether aneuploidy promotes or inhibits tumor growth, with lower rates of errors promoting tumor growth and higher rates leading to cell death and inhibiting tumor proliferation [50]. These studies provide a theoretical foundation for understanding the diverse functional regulatory roles of RIT1 during mitosis. By integrating these findings with our own research, we proposed that RIT1 is a key regulatory factor in mitosis and that its regulation of mitosis is likely a dynamic and coordinated process. Acetylation protection of SMC3 by RIT1 during mitosis may partially rescue mitotic errors caused by the negative regulation of SAC by RIT1, thereby ensuring efficient division and proliferation of tumor cells to drive tumor progression.

## Conclusions

In conclusion, our data demonstrate the critical role of RIT1 in HCC mitosis. Mechanistically, RIT1 regulates mitotic processes by interacting with SMC3 and PDS5, thereby protecting SMC3 acetylation and promoting rapid cell division and proliferation in HCC. These findings suggest that targeting RIT1 to induce mitotic catastrophe may be a promising therapeutic strategy for HCC.

### Electronic supplementary material

Below is the link to the electronic supplementary material.

**Supplementary Material 1: Figure S1.** The construction of RIT1 knockdown HCC cell lines. (A) Western blot analysis of RIT1 protein expression in HCC cell lines. (B) MHCC-97H and HCC-LY10 cells with silencing RIT1 expression were stably established by lentivirus transduction. The expression levels of RIT1 were verified by qPCR and western blot. **Figure S2.** Knockdown of RIT1 induces cell cycle arrest in HCC cells. (A) Flow cytometry analysis of cell cycle for MHCC-97H cells with RIT1 knockdown and control (left). The quantitation histogram showing the percentage of cells in different phases of the cell cycle (right). Data are presented as mean ± SD of three independent experiments. *** *P* < 0.001. The *P* values were calculated by unpaired Student’s *t* test. **Figure S3.** RIT1 concentrated around the chromosomes during mitosis. (A) Immunofluorescence staining of Myc-tag (red) and DAPI (blue) shows the distribution of RIT1 at different cell cycle phases in Hep3B cells transfected with Myc-RIT1 plasmid. Scale bars, 20 µm. **Figure S4.** RIT1-interacting protein screened by Co-IP and MS. (A) The Venn diagram of protein expression abundance in Myc-RIT1 group, which is three times higher than that of the IgG group in Huh7, HCC-LY10 and Hep3B cells. (B) Interaction analysis of SMC2, PLK1, and PRKDC with RIT1 in HCC-LY10 and Huh7 cells transfected with Myc-RIT1 plasmid by Co-IP and western blot. Cell lysates from indicated cells were immunoprecipitated using anti-Myc antibody. **Figure S5.** The co-localization of RIT1 and SMC3 during mitosis in Hep3B cells. (A) The co-localization of RIT1 and SMC3 during interphase and different phases of mitosis was analyzed by co-immunofluorescence staining (Myc-RIT1: red, SMC3: green, DAPI: blue) in Hep3B cells transfected with Myc-RIT1 plasmid. Scale bars, 20 µm. **Figure S6.** SMC3 is upregulated in HCC tissues and knockdown of SMC3 contributes to mitotic catastrophe. (A) Western blot analysis of protein expression of SMC3 in 36 paired HCC and adjacent non-cancerous tissues in our lab. (B) The fold changes of SMC3 protein expression levels in HCC tissues compared with adjacent non-cancerous tissues. (C) The protein expression and mRNA expression of SMC3 in HCC cell lines. (D) MHCC-97H and HCC-LY10 cells with silencing SMC3 expression were stably established by lentivirus transduction. The protein levels of SMC3 were verified by western blot. (E) The quantitation histogram of colony numbers for MHCC-97H and HCC-LY10 cells with SMC3 knockdown and control. (F) Representative immunofluorescence staining images of α-tubulin (red) and DAPI (blue) show multipolar spindles formation during mitosis in HCC-LY10 cells with SMC3 knockdown (left). The quantitation histogram showing the percentage of multispindle polar cells (right). (G) Representative immunofluorescence staining images of α-tubulin (red) and DAPI (blue) show multinucleated, heterogeneous nuclei cells in HCC-LY10 cells with SMC3 knockdown (left). The quantitation histogram showing the percentage of multinuclear cells (right).  Scale bars, 20 µm. Data are presented as mean ± SD of three independent experiments. *** *P* < 0.001. The *P* values were calculated by paired Student’s *t* test in (E). **Figure S7.** RIT1 does not affect the mRNA expression level of SMC3. (A) qPCR analysis of mRNA expression level of SMC3 in MHCC-97H and HCC-LY10 cells with RIT1 knockdown or control. (B) qPCR analysis of mRNA expression level of SMC3 in Huh7 and Hep3B cells with RIT1 overexpression or control. **Figure S8.** RIT1 does not affect the mRNA expression level of ESCO1. (A) qPCR analysis of mRNA expression level of ESCO1 in MHCC-97H and HCC-LY10 cells with RIT1 knockdown or control. (B) qPCR analysis of mRNA expression level of ESCO1 in Huh7 and Hep3B cells with RIT1 overexpression or control. **Figure S9.** RIT1 has no effect on PDS5 expression. (A) Western blot analysis of PDS5A and PDS5B protein in MHCC-97H and HCC-LY10 cells with RIT1 knockdown. (B) Western blot analysis of PDS5A and PDS5B protein in Huh7 and Hep3B cells with RIT1 overexpression. **Figure S10.** PDS5 does not affect the interaction of RIT1 with SMC3. (A) Western blot analysis of interaction of RIT1 with SMC3 in HCC-LY10 cells with PDS5 knockdown or control. **Table S1.** Target sequences of shRNA and siRNA. **Table S2.** The primer used for qPCR. **Table S3.** The Antibodies used for WB/IP in this study. **Table S4.** The Antibodies used for IF/IHC in this study

**Supplementary Material 2: Table S5.** The GSEA results of gene sets associated with RIT1 in the TCGA LIHC database

## Data Availability

All the presenting data are available within the article or supplementary files.

## Abbreviations

RIT1: Ras-like without CAAX 1
HCC: Hepatocellular carcinoma
TCGA: The Cancer Genome Altas
GSEA: Gene Set Enrichment Analysis
NC: Negative control
IF: Immunofluorescence
Co-IP: Co-immunoprecipitation
IHC: Immunohistochemical
GO: Gene Ontology
SMC3: Structural Maintenance of Chromosomes 3
PDS5A: PDS5 cohesin associated factor A
PDS5B: PDS5 cohesin associated factor B
ESCO1: establishment of sister chromatid cohesion N-acetyltransferase 1
SMC2: structural maintenance of chromosome 2
PLK1: polo like kinase 1
PRKDC: protein kinase, DNA-activated, catalytic subunit
