# Supplementary material for: RIT1 regulates mitosis and promotes proliferation by interacting with SMC3 and PDS5 in hepatocellular carcinoma
Source: J Exp Clin Cancer Res. 2023 Nov 29;42:326. doi: 10.1186/s13046-023-02892-x (PMC10685607; doi:10.1186/s13046-023-02892-x)
Supplement: Supplementary file 1 — Supplementary Material 1: Figure S1. The construction of RIT1 knockdown HCC cell lines. (A) Western blot analysis of RIT1 protein expression in HCC cell lines. (B) MHCC-97H and HCC-LY10 cells with silencing RIT1 expression were stably established by lentivirus transduction. The expression levels of RIT1 were verified by qPCR and western blot. Figure S2. Knockdown of RIT1 induces cell cycle arrest in HCC cells. (A) Flow cytometry analysis of cell cycle for MHCC-97H cells with RIT1 knockdown and control (left). The quantitation histogram showing the percentage of cells in different phases of the cell cycle (right). Data are presented as mean ± SD of three independent experiments. *** P < 0.001. The P values were calculated by unpaired Student’s t test. Figure S3. RIT1 concentrated around the chromosomes during mitosis. (A) Immunofluorescence staining of Myc-tag (red) and DAPI (blue) shows the distribution of RIT1 at different cell cycle phases in Hep3B cells transfected with Myc-RIT1 plasmid. Scale bars, 20 µm. Figure S4. RIT1-interacting protein screened by Co-IP and MS. (A) The Venn diagram of protein expression abundance in Myc-RIT1 group, which is three times higher than that of the IgG group in Huh7, HCC-LY10 and Hep3B cells. (B) Interaction analysis of SMC2, PLK1, and PRKDC with RIT1 in HCC-LY10 and Huh7 cells transfected with Myc-RIT1 plasmid by Co-IP and western blot. Cell lysates from indicated cells were immunoprecipitated using anti-Myc antibody. Figure S5. The co-localization of RIT1 and SMC3 during mitosis in Hep3B cells. (A) The co-localization of RIT1 and SMC3 during interphase and different phases of mitosis was analyzed by co-immunofluorescence staining (Myc-RIT1: red, SMC3: green, DAPI: blue) in Hep3B cells transfected with Myc-RIT1 plasmid. Scale bars, 20 µm. Figure S6. SMC3 is upregulated in HCC tissues and knockdown of SMC3 contributes to mitotic catastrophe. (A) Western blot analysis of protein expression of SMC3 in 36 paired HCC and adjacent non [file 13046_2023_2892_MOESM1_ESM.docx]

**
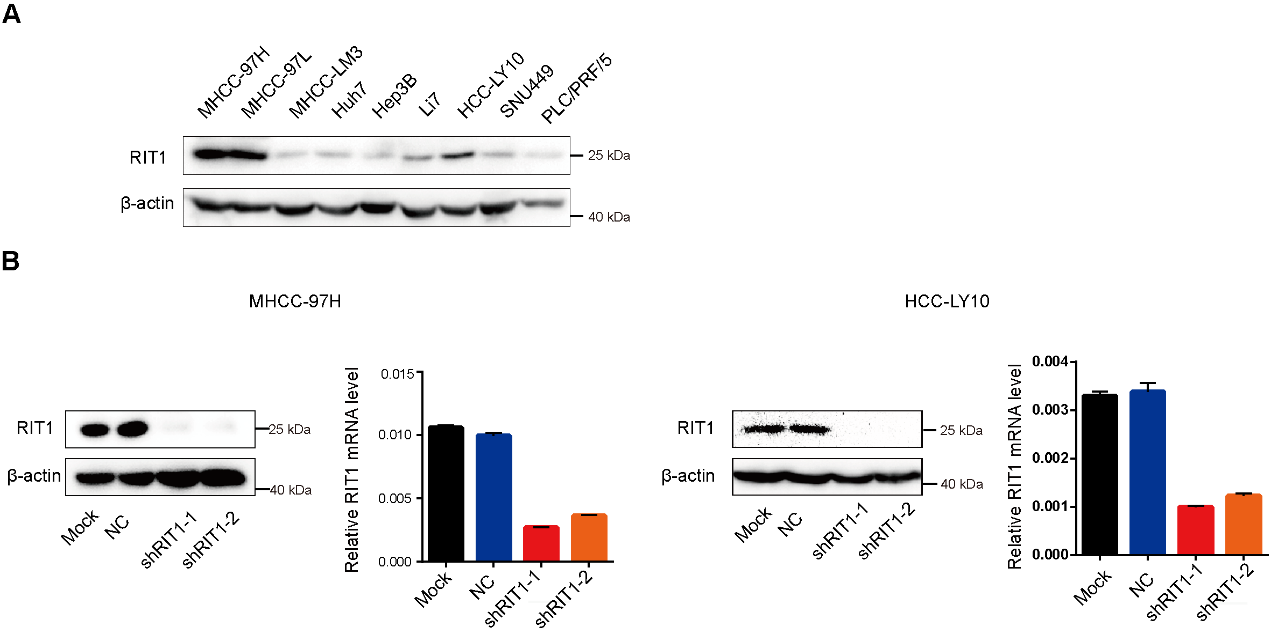
**

**Supplemental Figure S1** The construction of RIT1 knockdown HCC cell lines.

(A) Western blot analysis of RIT1 protein expression in HCC cell lines. (B) MHCC-97H and HCC-LY10 cells with silencing RIT1 expression were stably established by lentivirus transduction. The expression levels of RIT1 were verified by qPCR and western blot.


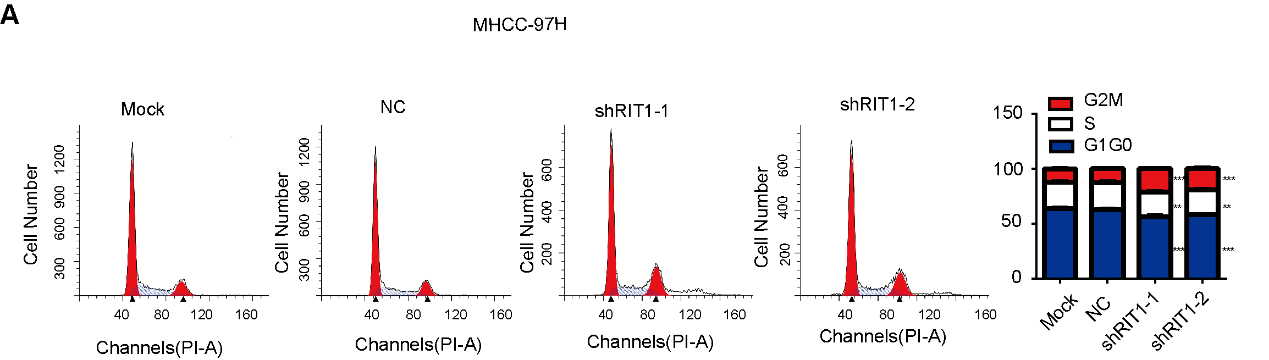


**Supplemental Figure** **S2** Knockdown of RIT1 induces cell cycle arrest in HCC cells.

(A) Flow cytometry analysis of cell cycle for MHCC-97H cells with RIT1 knockdown and control (left). The quantitation histogram showing the percentage of cells in different phases of the cell cycle (right). Data are presented as mean ± SD of three independent experiments. ** *P* < 0.01, *** *P* < 0.001. The *P* values were calculated by unpaired Student’s *t* test.


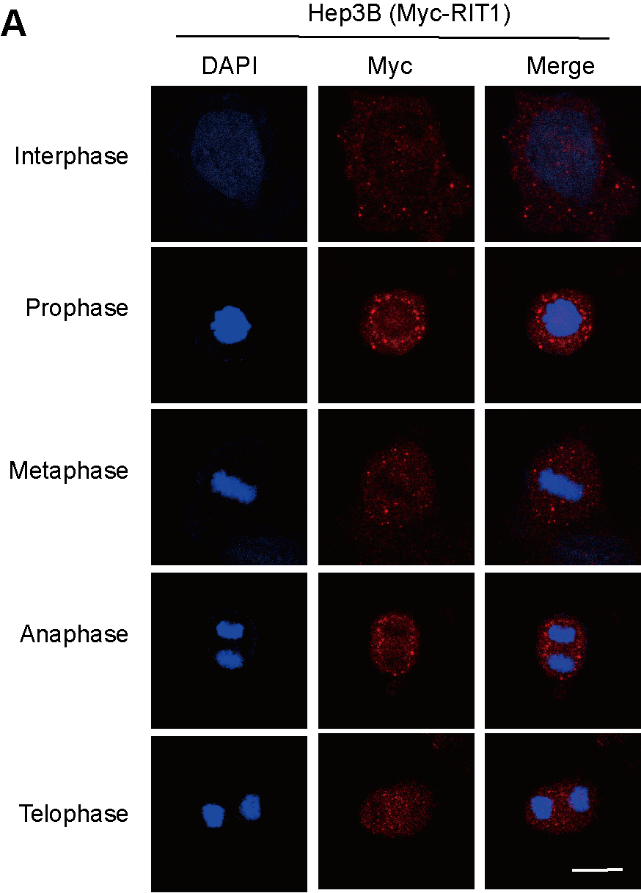


**Supplemental Figure S3** RIT1 concentrated around the chromosomes during mitosis. (A) Immunofluorescence staining of Myc-tag (red) and DAPI (blue) shows the distribution of RIT1 at different cell cycle phases in Hep3B cells transfected with Myc-RIT1 plasmid. Scale bars, 20 μm.

**
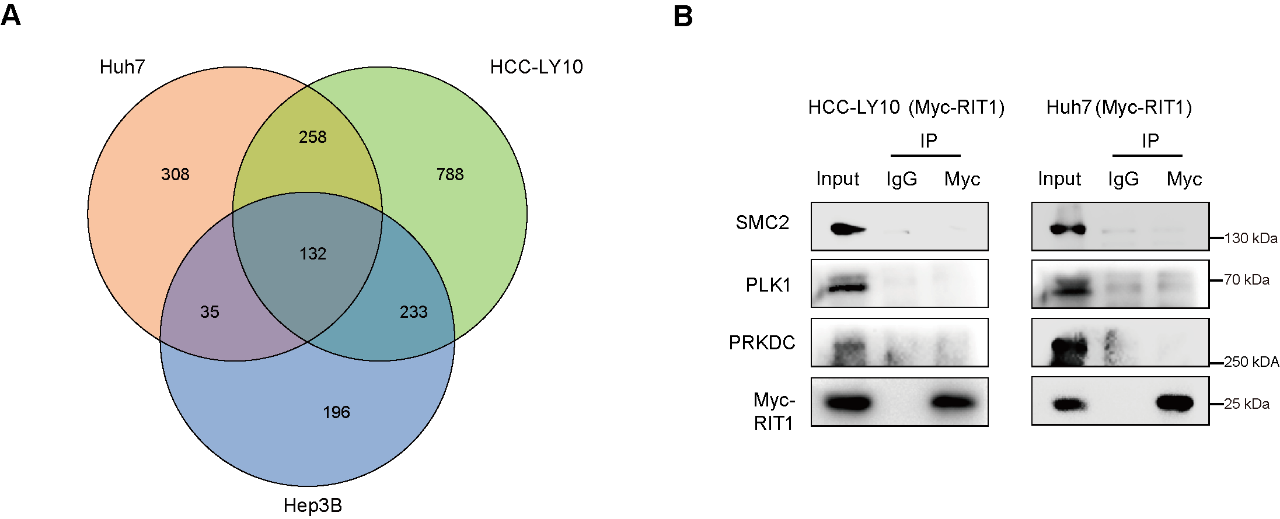
**

**Supplemental Figure S4** RIT1-interacting protein screened by Co-IP and MS.

(A) The Venn diagram of protein expression abundance in Myc-RIT1 group, which is three times higher than that of the IgG group in Huh7, HCC-LY10 and Hep3B cells.

(B) Interaction analysis of SMC2, PLK1, and PRKDC with RIT1 in HCC-LY10 and Huh7 cells transfected with Myc-RIT1 plasmid by Co-IP and western blot. Cell lysates from indicated cells were immunoprecipitated using anti-Myc antibody.


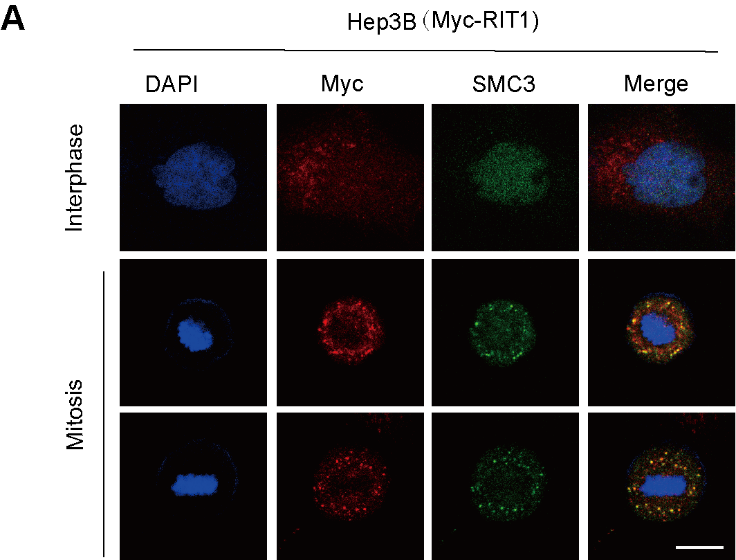


**Supplemental Figure S5** The co-localization of RIT1 and SMC3 during mitosis in Hep3B cells.

(A) The co-localization of RIT1 and SMC3 during interphase and different phases of mitosis was analyzed by co-immunofluorescence staining (Myc-RIT1: red, SMC3: green, DAPI: blue) in Hep3B cells transfected with Myc-RIT1 plasmid. Scale bars, 20 μm.


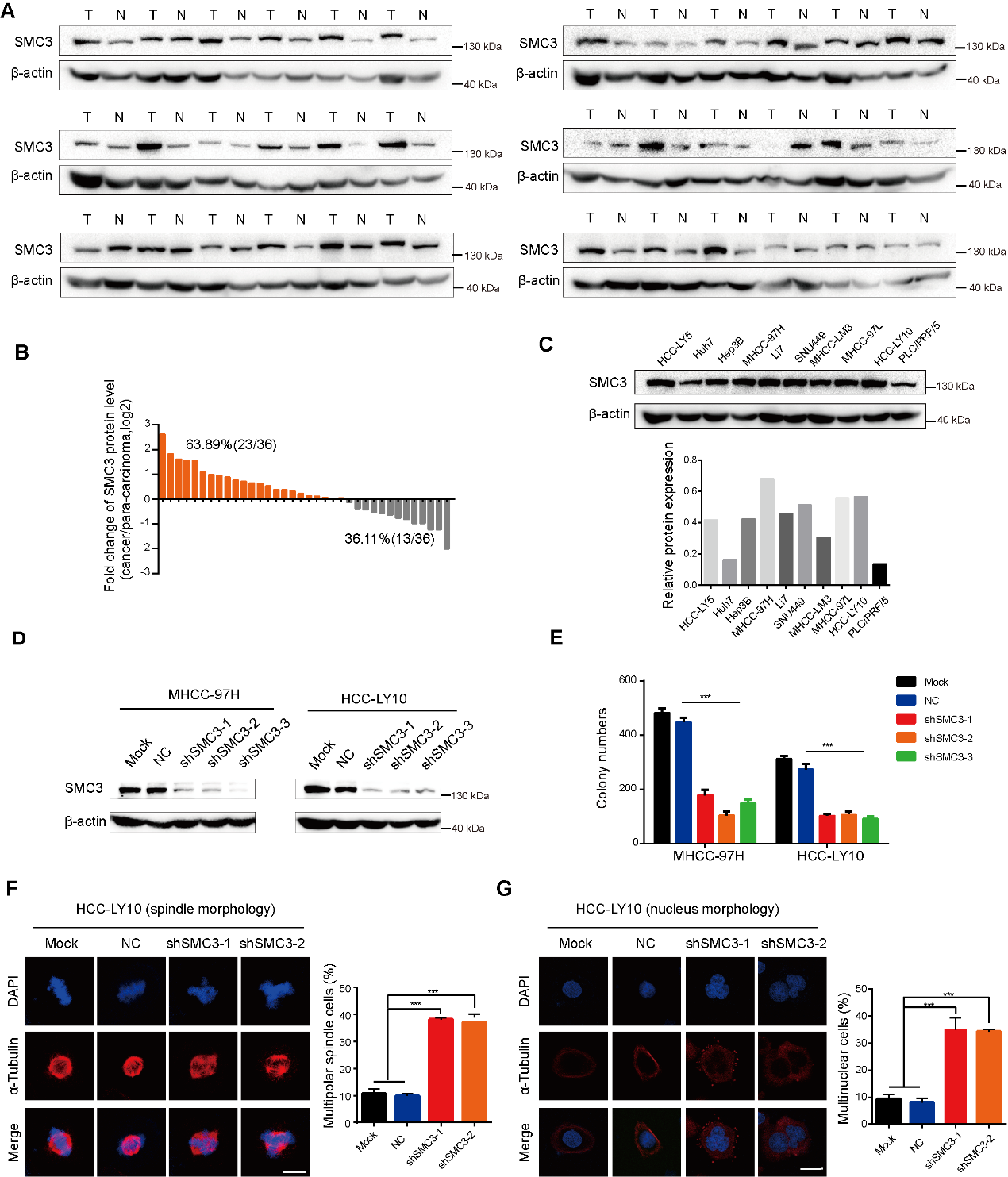


**Supplemental Figure S6** SMC3 is upregulated in HCC tissues and knockdown of SMC3 induces mitotic catastrophe.

(A) Western blot analysis of protein expression of SMC3 in 36 paired HCC and adjacent non-cancerous tissues in our lab. (B) The fold changes of SMC3 protein expression levels in HCC tissues compared with adjacent non-cancerous tissues. (C) The protein expression of SMC3 in HCC cell lines. (D) MHCC-97H and HCC-LY10 cells with silencing SMC3 expression were stably established by lentivirus transduction. The protein levels of SMC3 were verified by western blot. (E) The quantitation histogram of colony numbers for MHCC-97H and HCC-LY10 cells with SMC3 knockdown and control. (F) Representative immunofluorescence staining images of α-tubulin (red) and DAPI (blue) show multipolar spindles formation during mitosis in HCC-LY10 cells with SMC3 knockdown (left). The quantitation histogram showing the percentage of multispindle polar cells (right). (G) Representative immunofluorescence staining images of α-tubulin (red) and DAPI (blue) show multinucleated, heterogeneous nuclei cells in HCC-LY10 cells with SMC3 knockdown (left). The quantitation histogram showing the percentage of multinuclear cells (right). Scale bars, 20 μm. Data are presented as mean ± SD of three independent experiments. *** *P* < 0.001. The *P* values were calculated by unpaired Student’s *t* test in (E).

**
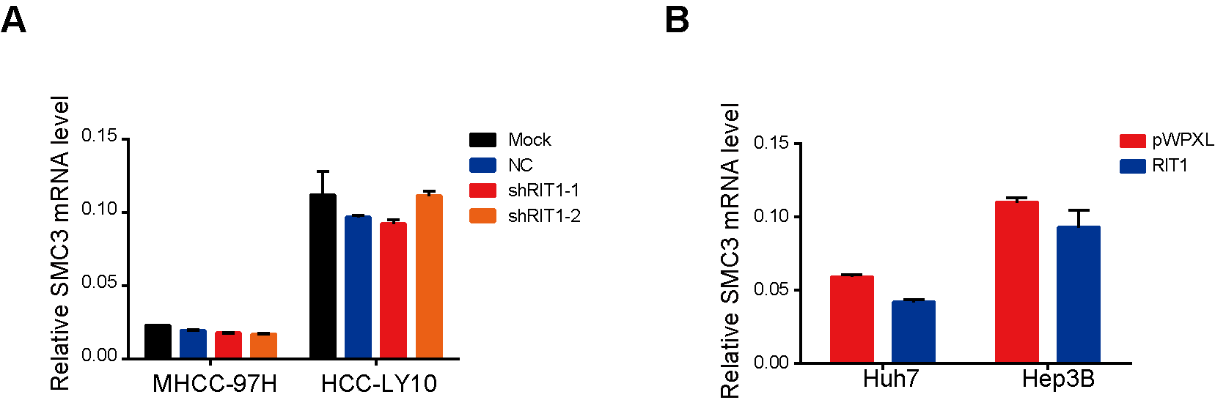
**

**Supplemental Figure S7** RIT1 does not affect the mRNA expression level of SMC3.

(A) qPCR analysis of mRNA expression level of SMC3 in MHCC-97H and HCC-LY10 cells with RIT1 knockdown or control. (B) qPCR analysis of mRNA expression level of SMC3 in Huh7 and Hep3B cells with RIT1 overexpression or control.


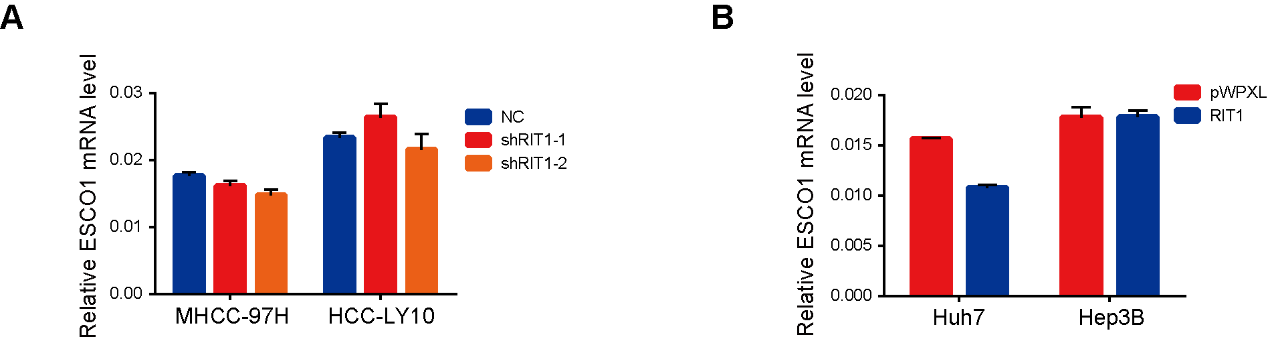


**Supplemental Figure S8** RIT1 does not affect the mRNA expression level of ESCO1.

(A) qPCR analysis of mRNA expression level of ESCO1 in MHCC-97H and HCC-LY10 cells with RIT1 knockdown or control. (B) qPCR analysis of mRNA expression level of ESCO1 in Huh7 and Hep3B cells with RIT1 overexpression or control.


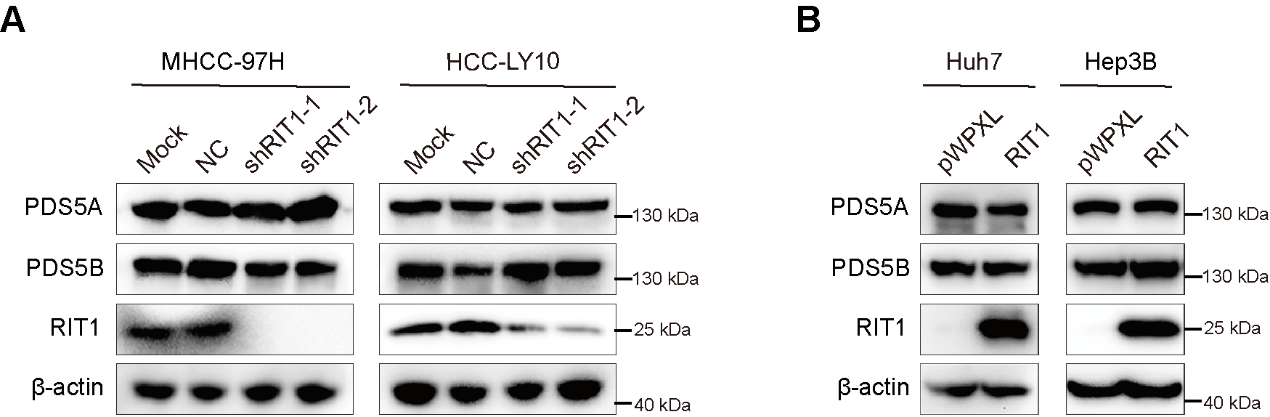


**Supplemental Figure S9** RIT1 has no effect on PDS5 expression.

(A) Western blot analysis of PDS5A and PDS5B protein expression in MHCC-97H and HCC-LY10 cells with RIT1 knockdown. (B) Western blot analysis of PDS5A and PDS5B protein expression in Huh7 and Hep3B cells with RIT1 overexpression.


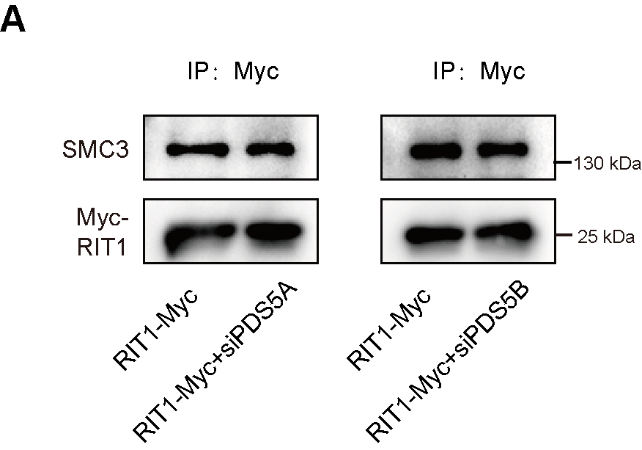


**Supplemental Figure S10** PDS5 does not affect the interaction of RIT1 with SMC3.

(A) Western blot analysis of interaction of RIT1 with SMC3 in HCC-LY10 cells with PDS5 knockdown or control.

**Supplemental Table S1** Target sequences of shRNA and siRNA

| **Name** | **Target sequence** |
| --- | --- |
| shNC-PLKO.1 | GACTATCATATGCTTACCGT |
| shRIT1-1 | CGTCGAAGTTTCCATGAAGTT |
| shRIT1-2 | CGAGAATTCAGCTGTCCCTTT |
| shSMC3-1 | GCAGTGCAACACAGAATTAAA |
| shSMC3-2 | CGAGTAGAGACTTATCTCAAT |
| shSMC3-3 | CCAAGTAGAACAGGAACTTAA |
| siESCO1-1 | GCTACAGAGTTATAGAAGA |
| siESCO1-2 | GTCAGATAATGTAGAGGTA |
| siPDS5A-1 | GCGATTAGCTGTTGTTCGA |
| siPDS5A-2 | GAACAGTCCAGACTATTGA |
| siPDS5B-1 | GAACCATCCTGATTTAGCA |
| siPDS5B-2 | GATGAATGCTATCAAGTAA |

**Supplemental Table S2** The primers used for qPCR

| **Primer Name** | **Primer Sequence** |
| --- | --- |
| RIT1-F | ACATCTGCTGCATACCGC |
| RIT1-R | TAGCCTCTTCCATACACTG |
| GAPDH-F | AGAAGGCTGGGGCTCATTTG |
| GAPDH-R | AGGGGCCATCCACAGTCTTC |
| SMC3-F | CAAGGAAAGATCAACCAGATGGC |
| SMC3-R | CCAGGGCTCGTCTCATTTTATC |
| ESCO1-F | TGCACGCAAGGATCTGAAAAG |
| ESCO1-R | CACTTGTGTATGAACCGACTTCT |

**Supplemental Table S3** The Antibodies used for WB/IP in this study

| **Antibody** | **Application** | **Dilution** | **Corporation** | **Catalogue** |
| --- | --- | --- | --- | --- |
| RIT1 | WB/IP | 1:1000; 1 ug/mg | Abcepta | AP51476 |
| SMC3 | WB/IP | 1:10000;2 ug/mg | Abcam | ab9263 |
| acetyl SMC3 | WB | 1:500 | Sigma-Aldrich | MABE1073 |
| Myc | WB/IP | 1:2000; 1 ug/mg | Sigma-Aldrich | 05-724 |
| Flag | WB/IP | 1:2000; 1 ug/mg | Sigma-Aldrich | F1804 |
| PRKDC | WB | 1:1000 | Proteintech | 19983-1-AP |
| SMC2 | WB | 1:1000 | Absin | Abs106086 |
| PLK1 | WB | 1:1000 | Proteintech | 12952-1-AP |
| PDS5A | WB | 1:2000 | Proteintech | 67520-1-Ig |
| PDS5B | WB | 1:2000 | Proteintech | 28318-1-AP |
| HRP-β-actin | WB | 1:25000 | Abcam | Ab8226 |
| HRP-anti-Rabbit IgG | WB | 1:5000 | CST | #7074 |
| HRP-anti-Mouse IgG | WB | 1:5000 | CST | #7076 |

**Supplemental Table S4** The Antibodies used for IF/IHC in this study

| **Antibody** | **Application** | **Dilution** | **Corporation** | **Catalogue** |
| --- | --- | --- | --- | --- |
| α-tubulin | IF | 1:1000 | Abcam | ab52866 |
| Myc Tag | IF | 1:100 | Sigma-Aldrich | 05-724 |
| SMC3 | IF/IHC | 1:1000 | Abcam | ab9263 |
| RIT1 | IHC | 1:10 | Sigma-Aldrich | HPA053249 |
| anti-Rabbit IgG, Alexa Fluor™ 546 | IF | 1:100 | Thermofish | A-11035 |
| anti-Mouse IgG, Alexa Fluor™ 546 | IF | 1:100 | Thermofish | A-11030 |
| anti-Rabbit IgG, Alexa Fluor™ 488 | IF | 1:100 | Thermofish | A-21206 |
